# Developing ZNF Gene Signatures Predicting Radiosensitivity of Patients with Breast Cancer

**DOI:** 10.1155/2021/9255494

**Published:** 2021-08-31

**Authors:** Derui Yan, Mingjing Shen, Zixuan Du, Jianping Cao, Ye Tian, Ping Zeng, Zaixiang Tang

**Affiliations:** ^1^Department of Biostatistics, School of Public Health, Medical College of Soochow University, Suzhou, China; ^2^Jiangsu Key Laboratory of Preventive and Translational Medicine for Geriatric Diseases, Medical College of Soochow University, Suzhou, China; ^3^Department of Thoracic Surgery, The Second Affiliated Hospital of Soochow University, Suzhou, China; ^4^School of Radiation Medicine and Protection and Collaborative Innovation Center of Radiation Medicine of Jiangsu Higher Education Institutions, Soochow University, Suzhou, China; ^5^Department of Radiotherapy & Oncology, The Second Affiliated Hospital of Soochow University, Suzhou, China; ^6^Department of Biostatistics, School of Public Health, Xuzhou Medical University, Xuzhou, China

## Abstract

Adjuvant radiotherapy is one of the main treatment methods for breast cancer, but its clinical benefit depends largely on the characteristics of the patient. This study aimed to explore the relationship between the expression of zinc finger (ZNF) gene family proteins and the radiosensitivity of breast cancer patients. Clinical and gene expression data on a total of 976 breast cancer samples were obtained from The Cancer Genome Atlas (TCGA) database. ZNF gene expression was dichotomized into groups with a higher or lower level than the median level of expression. Univariate and multivariate Cox regression analyses were used to evaluate the relationship between ZNF gene expression levels and radiosensitivity. The Molecular Taxonomy Data of the International Federation of Breast Cancer (METABRIC) database was used for validation. The results revealed that 4 ZNF genes were possible radiosensitivity markers. High expression of ZNF644 and low expression levels of the other 3 genes (ZNF341, ZNF541, and ZNF653) were related to the radiosensitivity of breast cancer. Hierarchical cluster, Cox, and CoxBoost analysis based on these 4 ZNF genes indicated that patients with a favorable 4-gene signature had better overall survival on radiotherapy. Thus, this 4-gene signature may have value for selecting those patients most likely to benefit from radiotherapy. ZNF gene clusters could act as radiosensitivity signatures for breast cancer patients and may be involved in determining the radiosensitivity of cancer.

## 1. Introduction

Breast cancer ranks as the fifth cause of cancer death overall and is the most commonly diagnosed cancer and the leading cause of cancer death in women [1, 2]. In 2018, there were about 2.1 million newly diagnosed female breast cancer patients worldwide, accounting for nearly a quarter of female cancer cases [2]. Treatment for breast cancer includes surgery, chemotherapy, and radiotherapy. A series of studies have confirmed that radiotherapy can effectively reduce the risk of local and overall recurrence and can improve overall survival [3–6]. Thus, after breast-conserving surgery, radiotherapy reduced the recurrence rate by half and mortality by about one-sixth [7]. However, not all breast cancer patients benefit from radiotherapy. Although well tolerated by most breast cancer patients, some patients may experience breast or chest wall pain during and after radiotherapy, and the known late side effects of radiotherapy include rib fractures, heart disease, and lymphedema [8, 9]. The absolute benefit of radiotherapy depends largely on the characteristics of the patient. Identifying factors related to individual radiosensitivity will optimize adjuvant radiotherapy and reduce the severity of normal tissue responses. Predicting tumor response to radiotherapy is one of the main problems of cancer treatment.

Until now, multigene signatures constructed based on mRNA [10, 11], lncRNA [12, 13], or miRNA [14] expression levels have been extensively studied to provide prognostic and predictive information for breast cancer treatment. For instance, the 9-mRNA signature can distinguish patients with different risks for overall survival and further predict the survival probability of patients [10]. The genome instability-derived lncRNA signature was used as an independent prognostic marker to stratify risk subgroups for patients with breast cancer [13]. A GI-derived three miRNA signature was used as a minimally invasive predictor of risk and unfavorable prognosis in breast cancer [14]. Most of the existing signatures can predict overall survival or local recurrence, but few signatures can predict the benefit of treatment. Although prognostic biomarkers can determine which patients will have a poor outcome and may require intensive treatment, they cannot determine which specific treatment should be given or whether the desired results can be obtained from these treatments. The development of predictive models has more clinical value [15].

Nowadays, many methods have been developed to combine gene therapy or molecular therapy with radiotherapy to predict which patients may be sensitive to and benefit from radiotherapy. Previous studies have shown that gene expression patterns can predict the intrinsic radiosensitivity of cells. Gene signatures have been identified and validated for predicting the radiosensitivity of many cancer types including gastric cancer [16], breast cancer [17], and glioma [18]. A 31-gene cluster related to cell cycle, cell adhesion, and cell junctions was found to have the potential to predict the radiosensitivity of cancer cells [19]. A radiosensitivity molecular signature [radiosensitivity index (RSI)] was clinically validated in 4 independent datasets (breast, rectal, esophageal, and head and neck) [20, 21]. Gene signatures can be used as predictive biomarkers to identify radiosensitive patients and optimize adjuvant radiotherapy.

Alterations in the transcriptome are a common feature of human cancer. In many cases, transcriptional disorders are caused by changes in expression levels or transcription factor activity [22]. Zinc finger (ZNF) proteins are the largest family of transcriptional regulators in mammals. The classic zinc finger protein family includes three categories, which are known as the BTB domain (Broad-complex, Tramtrack, and Bric-a-brac), the Krüppel-associated box (KRAB), and the newly defined SCAN domain, also known as the leucine-rich domain [23]. The functions of zinc finger proteins are extremely diverse, such as DNA recognition, RNA packaging, protein folding and assembly, lipid binding, transcriptional activation, and regulation of apoptosis [24–26]. Several family members mediate various different functions in tumor pathobiology. ZNF750 and ZNF545 have been identified as tumor suppressors [27, 28]. ZNF750 induces cell cycle arrest in the *G*0/*G*1 phase and regulates the tumor vascular microenvironment to inhibit oral squamous cell carcinoma's malignant progression. ZNF545 inhibits breast tumor cell proliferation by inducing apoptosis. Downregulation of ZNF121 and ZNF259 expression may inhibit cell proliferation and increase the proportion of apoptotic cells [29, 30]. The overexpression of ZNF genes is related to poor prognosis and lymph node metastasis [31–34]. Taken together, these studies indicate that ZNF genes may function as oncogenes involved in the occurrence and progression of cancer.

So far, no reports have appeared on correlations between ZNF genes and the radiosensitivity of breast cancer patients. In the present study, we used The Cancer Genome Atlas (TCGA) database to analyze the relationship between ZNF protein family genes and the radiosensitivity of breast cancer. Data from the Molecular Taxonomy of Breast Cancer International Consortium (METABRIC) database were employed for independent external validation. This study suggests new targets for the subsequent development of therapies for breast cancer.

## 2. Materials and Methods

### 2.1. Study Samples

**Figure 1:**
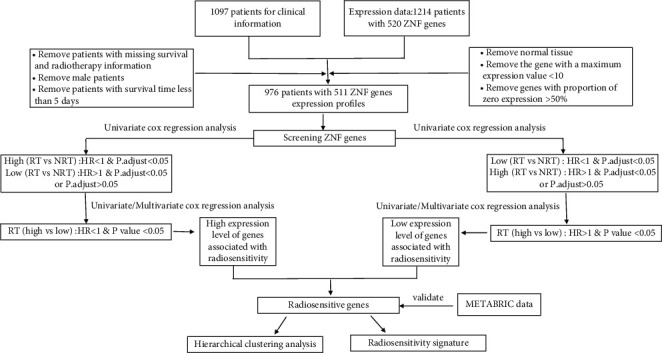
The workflow of data cleaning and analysis steps.

Clinical information and ZNF gene expression data of patients with breast cancer were downloaded from The Cancer Genome Atlas (TCGA; http://cancergenome.nih.gov/) via the R package TCGA assembler [35]. First, we eliminated patients without survival time data or survival status to select only those with clear survival information. In addition, we excluded samples of male breast cancer, patients with no radiotherapy data records, and those with less than 5 days of survival. Second, normalized read counts for the mRNA sequence data were downloaded. We eliminated genes with a maximum expression value <10 because they were barely expressed. Genes with zero expression rates in >50% of cases were also removed. Third, we merged the expression dataset and clinical dataset according to the patient's barcode number to obtain 511 gene expression profiles of 976 patients for the next phase of the analysis. The specific process of cleaning the clinical data is shown in Figure 1. Data from the Molecular Taxonomy of Breast Cancer International Consortium (METABRIC) database (http://www.cbioportal.org/) were obtained via the cBioPortal to perform the validation analysis. The GSE31863 dataset was available from the GEO database (https://www.ncbi.nlm.nih.gov/geo/).

### 2.2. Analysis Method

In the present study, radiosensitive patients were defined as a group of patients who had better overall survival after receiving radiotherapy compared with those who did not receive radiotherapy. Radiosensitivity genes were defined as those identifying patients responding to radiotherapy [16, 36]. ZNF genes were screened for further analysis by using two-step Cox regression analysis. The effect of radiotherapy on the overall survival of breast cancer was related to the expression levels of these ZNF genes. For example, if a gene related to radiosensitivity was expressed at a high level, patients who received radiotherapy had longer survival than patients who did not receive radiotherapy. For the low expression level of this gene, there were no significant survival differences between the two groups; thus, it is concluded that high expression of this gene was associated with radiosensitivity. The selected genes were determined based on the corresponding risk ratio and adjusted *P* value and those with significant interaction effects were selected for further analysis.

The distribution of expressed ZNF genes was skewed (Figure S1). According to the median expression value of each ZNF gene, the patients were divided into high and low expression subgroups. The Kaplan–Meier method was used to estimate survival time for the two subgroups, and the two-sided log-rank test was applied to determine statistical significance. Univariate and multivariate proportional hazard regression models were used to analyze the relationships between gene expression levels and radiosensitivity. Statistical analysis was performed in R version 3.6.3 using the packages “survival” and “rms.” The Benjamini–Hochberg method was used to perform multiple corrections. *P* < 0.05 was considered statistically significant. Missing values were multiply imputed using the R package “mice.” Hierarchical cluster analysis was performed by using R the package “heatmap.” The CoxBoost algorithm used the R package CoxBoost (https://github.com/binderh/CoxBoost) [37]. Random survival forests (RSF) algorithm used the R package “randomForestSRC.” The data analysis process is also summarized in Figure 1.

## 3. Results

### 3.1. Clinical Information and Survival Analysis

Clinical information on the 976 breast cancer patients included in the TCGA database is summarized in Table S1. Of these patients, 557 received radiotherapy. Multivariate analysis showed that receiving radiotherapy did not result in a statistically significant overall survival (OS) benefit when the whole patient cohort was analyzed (*P*= 0.068). The only clinical factors associated with the overall survival of these breast cancer patients were age (*P* < 0.001), pathological stage (*P* < 0.001), and having received chemotherapy (*P* = 0.025). A total of 1798 breast cancer patients is included in the METABRIC database, with 1094 patients receiving radiotherapy. After univariate and multivariate Cox regression analysis, radiotherapy was still related to the overall survival of breast cancer patients (Table S2). In the GSE31863 dataset, a total of 121 patients (66 patients receiving radiotherapy) were included in this study (Table S3). Univariate Cox regression analysis showed that there was no significant correlation between radiotherapy and the OS of breast cancer patients (*P* = 0.127), but in multivariate Cox regression analysis, radiotherapy showed a survival benefit (*P* = 0.03).

### 3.2. Radiosensitive ZNF Gene Development

Based on the detailed procedure shown in Figure 1, we analyzed the effects of radiotherapy on the overall survival of breast cancer patients with high and low levels of ZNF gene expression. According to the corresponding risk ratio and adjusted *P* value, the high expression of 253 ZNF genes and the low expression of a further 106 ZNF genes were informative for the outcome of radiotherapy. Of these, the expression levels of 51 genes were related to the overall survival of breast cancer patients receiving radiotherapy (Tables S4 and S5). There were 40 overlapping ZNF genes in the METABRIC database for validation. Univariate Cox regression analysis showed that the expression levels of 7 genes were related to radiosensitivity (Table S6). Multivariate Cox regression analysis of the two databases showed that high expression of ZNF644 and low expression of ZNF341, ZNF541, and ZNF653 were associated with radiosensitivity of breast cancer.

**Figure 2:**
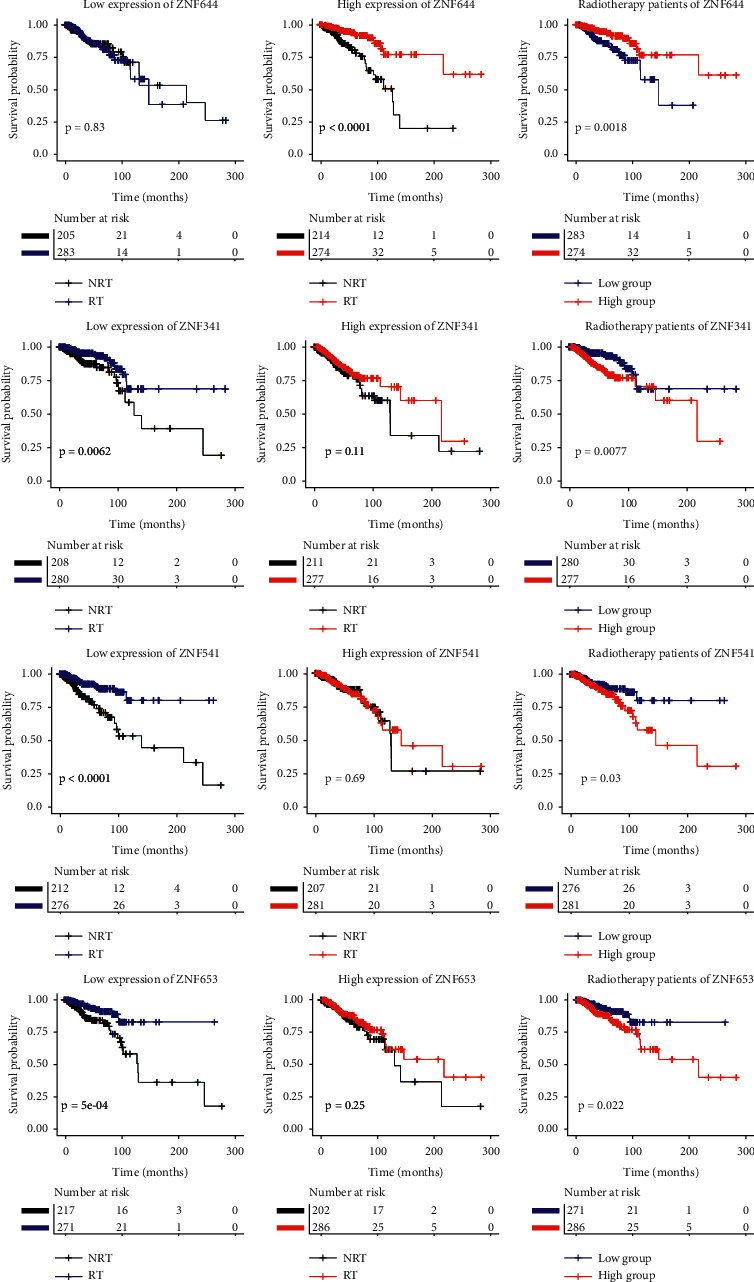
The survival curves comparison of radiotherapy and ZNF gene expression levels in TCGA data. RT, radiotherapy; NRT, nonradiotherapy.

**Figure 3:**
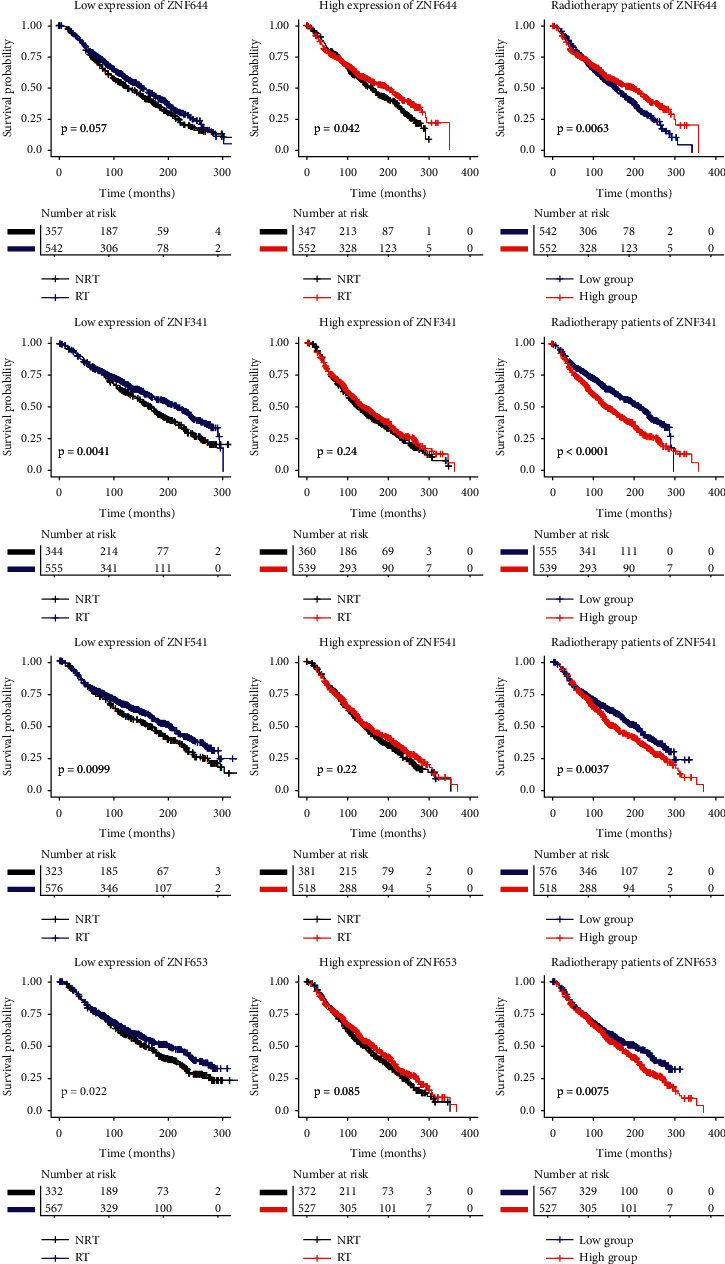
The survival curves comparison of radiotherapy and ZNF gene expression levels in METABRIC data. RT, radiotherapy; NRT, nonradiotherapy.

Figure 2 depicts a comparison of the Kaplan–Meier curves between radiotherapy and gene expression levels. When ZNF644 was expressed at a high level, patients who received radiotherapy had a longer survival time than those who did not receive radiotherapy. However, in patients with low ZNF644 expression, there were no significant survival differences between those receiving or not receiving radiotherapy. Among patients who did receive radiotherapy, those with high ZNF644 expression had significantly longer survival than patients with low level expression. For the other 3 genes, the results were the opposite; i.e., high expression resulted in no significant survival differences between patients receiving or not receiving radiotherapy but when these genes were expressed at low levels, patients receiving radiotherapy had a survival advantage. Thus, of patients receiving radiotherapy, those with low expression of these genes had significantly longer survival than patients with high expression. Figure 2 shows the results of univariate analysis of the TCGA data, and Figure 3 shows the results of univariate analysis of the METABRIC data which are consistent with these. Thus, similar conclusions can be drawn from the analyses in Figures 2 and 3.

**Figure 4:**
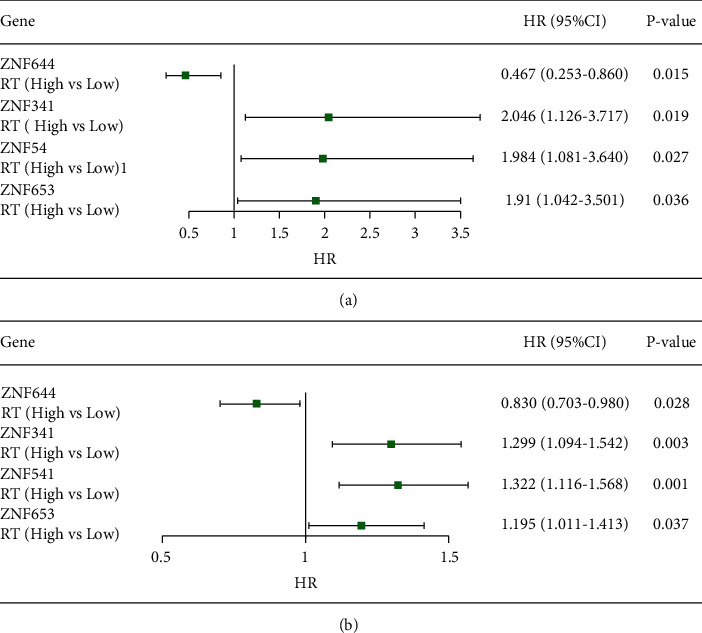
The HR estimation for high expression (High) vs. low expression (Low) of ZNF genes in the multivariable Cox regression models of radiotherapy population. (a) TCGA data, the adjusted factors are age, pathological stage, histological type, ER, PR, HER, and chemotherapy. (b) METABRIC data, the adjusted factors are age, grade, histological type, ER, PR, HER, and chemotherapy.

Multivariate analysis was also performed adjusting for age, pathological stage, ER, PR, HER, histological type, and chemotherapy (Figure 4). The four identified ZNF genes with the potential to select patients who are most likely to benefit from radiotherapy were shown to be independent of clinical variables.

### 3.3. Correlation of ZNF Radiosensitive Genes

**Figure 5:**
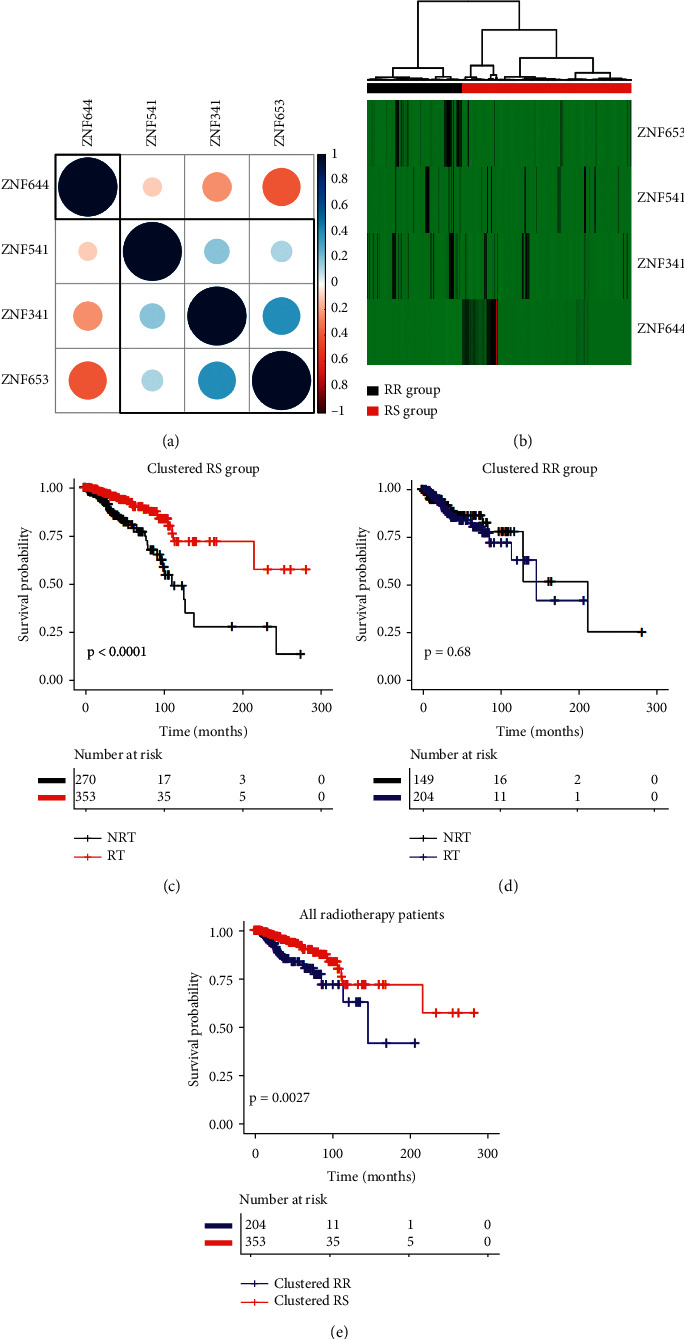
TCGA data. (a) Correlation plot based on Pearson's correlation test results to show the correlation of gene expression among the 4 ZNF family members. (b) Hierarchical clustering analysis. The top red and black bands denote the clustered radiosensitive (RS) and radioresistant (RR) patients, respectively. (c–e) The survival curves under radiotherapy and nonradiotherapy for both clustered radiosensitive (RS) and radioresistant (RR) patients.

**Figure 6:**
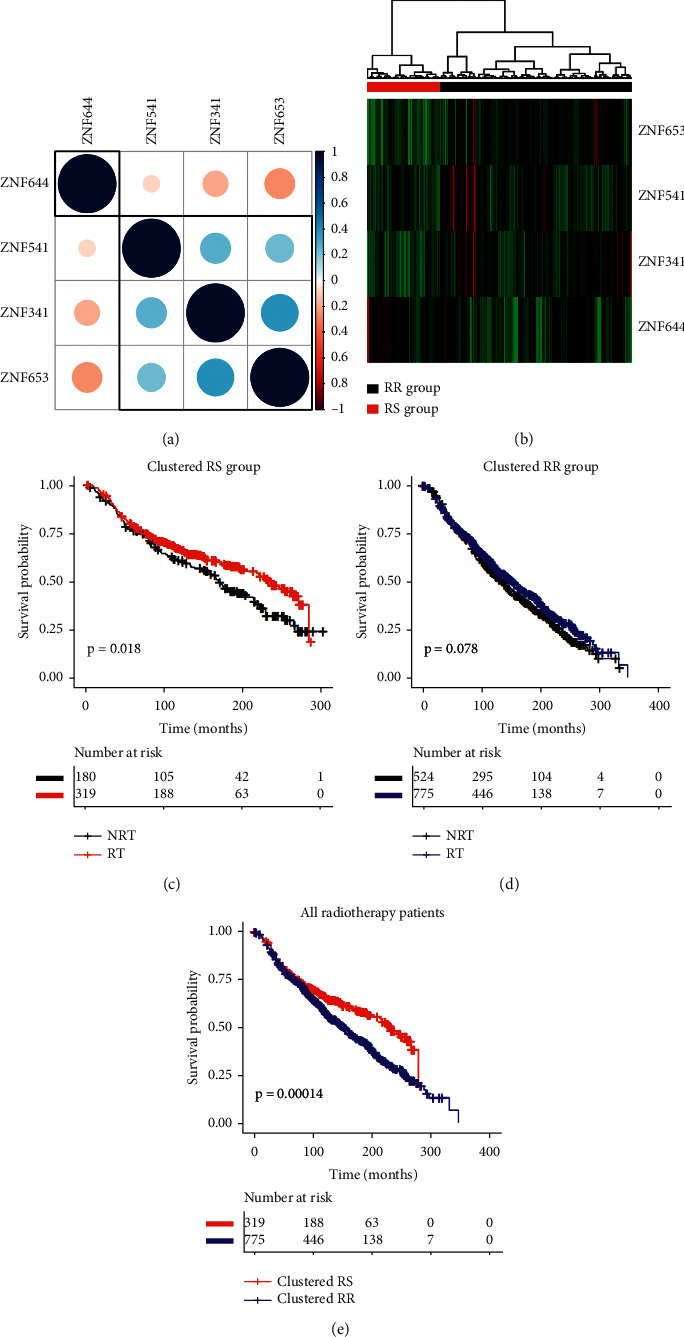
METABRIC data. (a) Correlation plot based on Pearson's correlation test results to show the correlation of gene expression among the 4 ZNF family members. (b) Hierarchical clustering analysis. The top red and black bands denote the clustered radiosensitive (RS) and radioresistant (RR) patients, respectively. (c–e) The survival curves under radiotherapy and nonradiotherapy for both clustered radiosensitive (RS) and radioresistant (RR) patients.

The results of the correlation analysis are depicted in Figures 5(a) and 6(a) showing an interesting correlation pattern. ZNF644 expression was negatively correlated with the other 3 genes (ZNF341, ZNF541, and ZNF653), which were positively correlated with each other.

### 3.4. Cluster and Survival Analysis

We further extracted expression patterns of these 4 genes for hierarchical cluster analysis (Figure 5(b)). The patients were classified into two groups according to this hierarchical cluster analysis. The red bar represents clustered radiosensitive (RS) patients (*n* = 623), and the black bar represents clustered radioresistant (RR) patients (*n* = 353). Patients in the RS group had significantly longer survival after receiving radiotherapy than patients who did not receive radiotherapy (*P* < 0.001) (Figure 5(c)). In contrast, radiotherapy did not improve the overall survival in the RR group (*P* = 0.68) (Figure 5(d)). Among the patients receiving radiotherapy, the survival of patients in the RS group was significantly longer than that in the RR group (*P* = 0.003) (Figure 5(e)). The results of the hierarchical cluster and univariate Cox regression analysis of the validation data are shown in Figure 6. Subsequently, multivariate analysis was further performed to assess the effect of radiotherapy on overall survival for the clusters of RS and RR patients.  Table 1 summarizes the raw and adjusted HR and *P* values by univariate and multivariate Cox regression analysis. Similarly, the univariate and multivariate Cox regression analysis results of the METABRIC data were consistent with the above outcome. Patients in the GSE31683 dataset were separated into clustered RS and RR groups (Figure S3). In the cluster RS group, patients who received radiotherapy tended to have better recurrence-free survival than those who did not (*P* = 0.045). By contrast, radiotherapy did not show an RFS benefit in the clustered RR group (*P* = 0.6). However, there was no significant improvement in RFS after radiotherapy in the RS group (*P* = 0.32).

### 3.5. Cox Prediction Model

**Figure 7:**
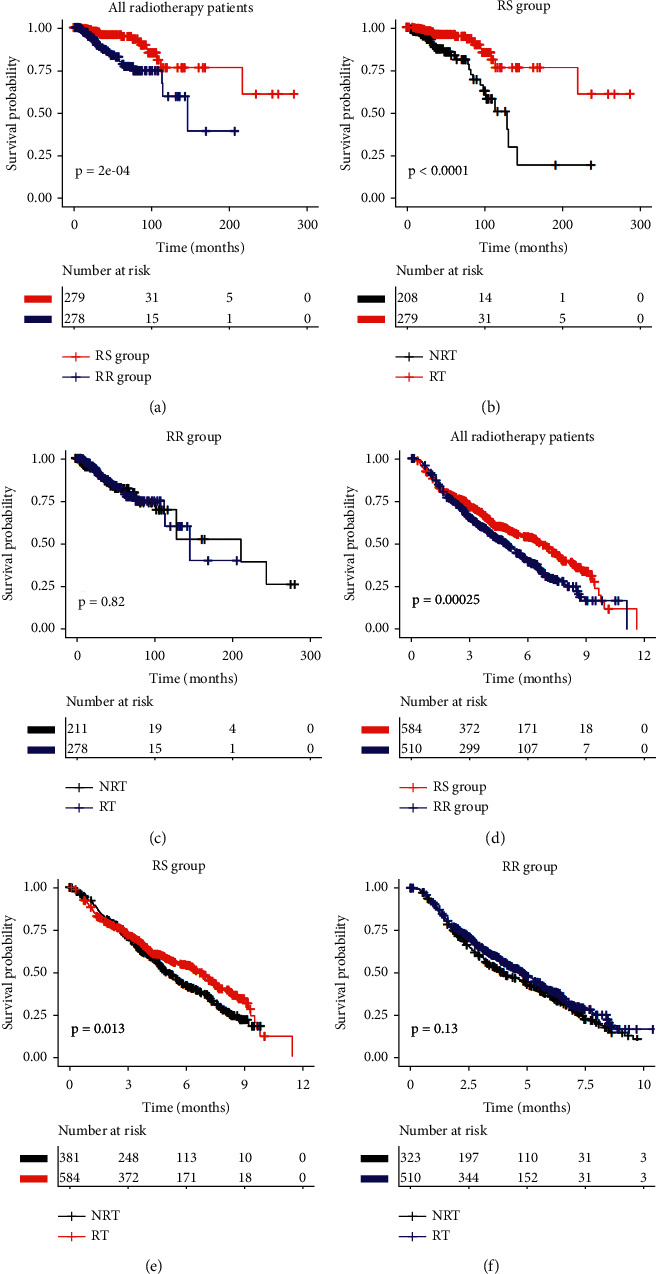
Overall survival stratified by Cox prediction model. The survival curves comparison of radiotherapy and 4-gene-based signature in (a–c) TCGA data and (d–f) METABRIC data. RT, radiotherapy; NRT, nonradiotherapy; RS, radiosensitive; RR, radioresistant.

**Figure 8:**
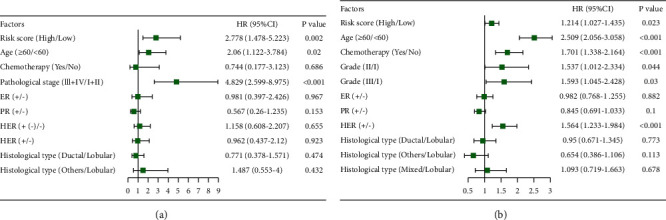
Forest plot of hazard ratios. Multivariable Cox regression analyses of the risk score, age, pathological stage (grade), histological type, ER, PR, HER, and chemotherapy in TCGA data (a) and METABRIC data (b). The blue squares represent the hazard ratio (HR). 95% CI, 95% confidence interval.

Among patients with TCGA data who received radiotherapy, a 4-mRNA ZNF gene signature comprising ZNF644, ZNF341, ZNF541, and ZNF653 was established. Thus, the risk score = (−0.61414^*∗*^ expression level of ZNF644) + (0.21667^*∗*^ expression level of ZNF341) + (−0.14907^*∗*^ expression level of ZNF541) + (−0.06819^*∗*^ expression level of ZNF653). The coefficients were assessed by multivariate regression analysis based on these 4 genes (Table S6). Patients could be divided into low- and high-risk groups based on the median risk score. Hence, the low-risk group was classified as the RS group, which had a higher survival rate after radiotherapy, while the high-risk group was classified as the RR group, which had a poorer outcome after radiotherapy (Figure 7(a)). In the RS group, patients who received radiation therapy had better overall survival than those who did not (Figure 7(b)). In contrast, radiotherapy did not improve overall survival in the RR group (Figure 7(c)). METABRIC data were used to validate this signature, with the result shown in Figures 7(d)–7(f). Multivariate Cox regression analysis showed that this signature was a predictive factor independent of other clinical factors (Figure 8). According to Cox regression analysis, the significant interaction between this 4-gene-based signature and radiotherapy indicates that it may have predictive value for identifying those patients who will benefit from radiotherapy. Patients in the GSE31863 dataset were separated into RS and RR groups using the cut-off established above (Figure S4). In the RS group, the RFS of patients who received radiotherapy did not improve significantly compared with patients who did not receive radiotherapy (*P* = 0.082). Besides, among radiotherapy patients, the RFS of RS patients was not significantly higher than that of RR patients (*P* = 0.73). KM plot showed that this gene signature did not have significant interaction with radiotherapy for predicting RFS.

### 3.6. CoxBoost Prediction Model

CoxBoost is an algorithm that allows the implementation of boosting in conjunction with the Cox model [38]. We used the “CoxBoost” package to fit the Cox model (tune by CoxBoost (): step. no = 100; penalty = 100). The coefficients of the selected multivariate Cox model were set by the last step of CoxBoost (Figure S5). Similarly, based on a combination of the gene coefficients and gene expression count, the risk score of each patient was calculated. Patients were divided into low- and high-risk groups based on the median risk score. Among radiotherapy patients, the prognosis of RS patients was significantly better than that of RR patients (Figures S6(a) and S6(d)). In the classed RS group, patients who received radiotherapy had a longer OS rate than those who did not receive radiotherapy (Figures S6(b) and S6(e)), but no significant difference was observed in the classed RR group (Figures S6(c) and S6(f)). Multivariate Cox regression analysis showed that this signature was a predictive factor independent of other clinical factors (Figure S7). The results of the CoxBoost model were similar to the results of the Cox model. KM plot showed that this gene signature did not have significant interaction with radiotherapy for predicting RFS (Figure S8).

### 3.7. Random Forests Prediction Model

To find out how useful these genes actually are for the prediction of survival after radiotherapy, we constructed a random survival forest model based on these four genes to predict the survival of patients after radiotherapy. As shown in Figure S9, the model was symbiosis into 500 binary survival trees, and each survival tree had 20 terminal nodes on average. The out-of-bag error to verify and predict the survival outcome of the model was 33.24%. The model was used to predict the survival outcome of the METABRIC dataset, and the error rate is 50.24%. This model was used to predict the recurrence of patients in the GSE31863 dataset after radiotherapy, with an error rate of 54.54%.

### 3.8. Comparison with Previously Published Gene Signatures

We compared two existing gene signatures for predicting radiosensitivity, a 31-gene cluster and the radiation sensitivity index (RSI), in the TCGA and METABRIC datasets. Neither 31-gene signature nor RSI showed a significant interaction with radiotherapy in the two datasets. Using hierarchical clustering based on the gene expression profile of the 31-gene signature, we divided the total patients into two clusters (Figures S10 and S11). Patients in the red bar were assigned to the RS group, and the survival rate of patients after radiotherapy was higher than that of patients without radiotherapy. Conversely, in the RR groups, there was no significant difference in survival rate between the radiotherapy and nonradiotherapy groups. However, among radiotherapy patients, the survival rate of clustered RS patients was not significantly better than that of clustered RR patients.

For the RSI, the 25th percentile of RSI in patients receiving radiotherapy was used as the cut-off value for dividing patients into RS and RR groups, as in previous studies [21]. RSI did not show significant results in predicting radiotherapy benefits in either the TCGA or METABRIC datasets (Figure S12).

## 4. Discussion

Radiotherapy is one of the main methods of breast cancer treatment, which has made substantial progress over the years with the development of improved treatment plans and implementation methods. However, through multivariate Cox regression analysis, we found that radiotherapy was not an important clinical factor related to the overall survival of the whole cohort of breast cancer patients in the TCGA dataset. However, it was significantly associated with overall survival in the METABRIC validation data. And, radiotherapy was significantly associated with recurrence-free survival in the GSE31863 dataset. Therefore, we concluded that not all patients can benefit from radiotherapy, nor is the absolute benefit equal across risk groups. Due to differences in tumor radiosensitivity, the response of cancer patients to radiotherapy varies greatly, while serious adverse consequences are universal. Therefore, the most important issue in this respect is to be able to accurately predict which patients will benefit from radiotherapy before beginning treatment. In the era of precision medicine, exploring the radiosensitivity of patients at the genetic level has attracted widespread attention. Cancer risk and radiosensitivity are commonly associated with deficiencies in DNA-damage recognition, DNA repair, cell cycle checkpoint activation, stress responses and signal transduction, premature senescence pathways, and/or apoptosis [39, 40]. It is known that hundreds of gene products are involved in determining the radiosensitivity of cells, and this number is still increasing as data are generated [40]. The zinc finger protein gene family is one of the largest human gene families and plays an important role in transcriptional regulation. In this study, we explored whether and which ZNF genes may be useful as radiosensitivity signatures and for personalized medical decisions.

In the present study, we analyzed female breast cancer patient clinical and gene expression information from the TCGA database. A total of 511 ZNF genes and 976 patients was involved in our initial analysis. First, ZNF genes were screened by using a two-step Cox regression analysis. The main idea of this step was to determine whether patients with lower or higher expression of ZNF genes were differently sensitive to radiotherapy. Next, univariate and multivariate Cox regression analysis was used to analyze the relationship between ZNF gene expression and overall survival in the patient cohort receiving radiotherapy. The results of TCGA and METABRIC data analyses indicated that ZNF gene signatures had statistically significant interactions with radiotherapy independent of clinical variables. These selected ZNF genes revealed that the effect of radiotherapy on patients is different according to their different levels of expression. It was assumed that some of the patients express several of these sensitivity marker genes, and those patients would be expected to have a relatively high probability of survival. They are referred to here as radiosensitive patients, and accordingly, the sensitivity genes were designated as providing a radiosensitive gene signature. We have identified a total of 4 ZNF genes related to the radiosensitivity of breast cancer. High expression of ZNF644 might be associated with the radiosensitivity of breast cancer patients, and reciprocally, low expression of the other 3 genes (ZNF341, ZNF544, and ZNF653) marked patients in the radiosensitive group. We found crossing survival curves with significant *P* values, e.g., survival curves for the gene ZNF341 (Figure 2). Landmark analyses were performed according to a landmark point at 112 months, with the KM survival analysis for events that occurred up to 112 months and events that occurred between 112 months and the end of the follow-up period.  Figure S2 shows the results of the landmark analyses of the overall survival and its components. Among patients receiving radiotherapy, patients with low expression of ZNF341 had significantly longer survival than patients with a high expression before the landmark point of 112 months. After the landmark point of 112 months, the survival rate of the low expression group was higher than that of the high expression group, but the difference was not statistically significant. The predictive value of longer-term radiosensitivity according to ZNF341 expression needs further study.

To explore the potential relationship between the radiosensitivity-associated genes, correlation coefficients between the 4 ZNF genes were calculated. These results indicated that the 3 genes may share some common features or functions, and the 4 ZNF genes may have a synergistic prognostic effect. Due to the limited information available, it has not been proven possible to establish transcriptional regulatory networks for these four genes. The result of hierarchical cluster analysis showed that all breast cancer patients could be divided into two groups according to their expression of these 4 genes. Patients clustered as radiosensitive had significantly longer survival when compared with nonradiosensitive patients. Thus, the 4-mRNA ZNF signature comprising ZNF644, ZNF341, ZNF541, and ZNF653 was established. Cox regression and CoxBoost analysis showed that there was a significant interaction between this signature and the overall survival of radiotherapy. The random survival forest model also showed that these four genes could predict the survival status of patients after radiotherapy. We used clustering, Cox modeling, CoxBoost modeling, and random survival forest methods to demonstrate that these four genes have the value of predicting radiosensitivity in breast cancer patients. These radiosensitivity marker genes were designed to be predictive because they were identified in patients who received radiation therapy, using an appropriate control group of patients who did not receive radiotherapy. Furthermore, in the GSE31863 dataset, the RS patients did not show significantly longer recurrence-free survival than the RR patients. However, in the cluster RS group, the radiotherapy patients had significantly longer recurrence-free survival than the nonradiotherapy patients. In the RS group, recurrence-free survival was longer in radiotherapy patients than in nonradiotherapy patients, although no significant difference was achieved. However, this still provides evidence for the radiosensitivity study of breast cancer recurrence-free survival. Due to the limited sample size of GSE31863, the extrapolation of this gene signature to predict other outcomes of radiosensitivity requires further research. In addition, by comparing with the previously developed radiosensitivity gene signatures, we found that this ZNF signature has obvious advantages. In both clustering and modeling, the overall survival of RS patients after radiotherapy was significantly higher than that of RR patients after radiotherapy. There was a significant interaction between the 4-gene signature and radiotherapy according to the Cox regression analysis, suggesting that the ZNF signature could have predictive value. This ZNF signature has the potential to select patients who are most likely to benefit from radiotherapy.

The zinc finger protein (KRAB-ZFP) family accounts for at least one-third of mammalian ZNF proteins. KRAB-ZFP contains an effector motif denoted the Krüppel-associated box or KRAB, which seems to be unique to vertebrates [23, 41, 42]. The KRAB-ZNF family is very complex, containing a large number of homologous genes, gene isoforms, and pseudogenes [26]. There are more than 800 ZNF genes in the human genome, including 8000 ZNF domains. Interestingly, approximately 266 of these genes are found on chromosome 19, of which 202 are KRAB-ZNF genes [43]. ZNF541 and ZNF653 are both located on chromosome 19. Although all chromosomes are unique, chromosome 19 can be considered unusual in that it possesses the highest gene density of all human chromosomes, more than twice the average for the whole genome. Studies have found that gene mutations on chromosome 19 are associated with the occurrence of malignant tumors [44]. There are few reports on the biological functions of ZNF541 and ZNF653. Some studies have pointed out that the aberrant expression of ZNF644 may be a potential pathogenic factor for rheumatoid arthritis and strong myopia [45, 46].

ZNF341, a transcription factor with 12 Cys2His2 zinc fingers, regulates STAT3 (signal transducer and activator of transcription 3) and many other genes, including STAT1, because it can bind to the STAT3 promoter and activate the transcription of the STAT3 reporter plasmid [47]. STAT3 is a central regulator of tumor immune tolerance. Importantly, STAT3 can act as a negative feedback regulator, and targeted inhibition of STAT3 can enhance the efficacy of radiotherapy for head and neck cancer [48], lung cancer [49], and breast cancer [50]. Studies have shown that transcription factor ZNF341 is a positive regulator of STAT3 expression [51]. It is known that ZNF341 transcriptionally regulates the expression of STAT3 which seems to be necessary for the efficient repair of damaged DNA. Therefore, based on Comet assay results, it was hypothesized that increased radiosensitivity was due to the lack of efficient DNA repair [52]. Further studies are needed to better understand how ZNF341 regulates radiotherapy responses.

The main limitation of our study is the use of retrospective cohorts. We also need some related experiments to verify these gene signatures. Ideally, predictive biomarkers should be tested in prospective randomized controlled trials. However, we have verified these sensitivity signatures in two large retrospective databases, which provide directions for follow-up clinical studies. There are no reports on associations between ZNF genes and radiosensitivity of breast cancer patients to date. Therefore, the mechanisms of ZNF genes and radiosensitivity of breast cancer need further study.

## 5. Conclusion

Associations between cancer radiosensitivity and ZNF family gene expression have not been reported before. In the present study, we explored this association by using the TCGA and METABRIC data. According to our results, patients with high expression of ZNF644 were sensitive to radiotherapy, while low expression of ZNF314, ZNF544, and ZNF653 marked those who were radiosensitive. Although the biological mechanism responsible for these findings is not clear, and further work is necessary, at least the present study suggests that these 4 ZNF genes could act as radiosensitivity signatures for breast cancer patients. Our study provides important clues for determining the radiosensitivity of breast cancer and also offers a new avenue for clinical improvement in breast cancer treatment.

## Figures and Tables

**Table 1:** Hierarchical clustering analyses.

		Raw		Adjusted	
		HR (95% CI)	*P* value	HR (95% CI)	*P* value
TCGA data					
	Clustered RS (RT/NRT)	0.346 (0.210–0.570)	<0.001	0.253 (0.147–0.438)	<0.001
	Clustered RR (RT/NRT)	1.143 (0.612–2.135)	0.676	1.411 (0.682–2.918)	0.353
	RT patients (RS/RR)	0.436 (0.250–0.762)	0.004	0.437 (0.242–0.788)	0.006

METABRIC data					
	Clustered RS (RT/NRT)	0.734 (0.567–0.950)	0.019	0.650 (0.491–0.862)	0.003
	Clustered RR (RT/NRT)	0.882 (0.767–1.014)	0.078	0.868 (0.751–1.004)	0.056
	RT patients (RS/RR)	0.687 (0.565–0.835)	<0.001	0.741 (0.606–0.907)	0.004

The adjusted factors included age, pathological stage (grade), histological type, ER, PR, HER, and chemotherapy. HR, hazard ratio; 95% CI, 95% confidence interval; RT, radiotherapy; NRT, nonradiotherapy; RS, radiosensitive; RR, radioresistant.

## Data Availability

The datasets used in the present study are available from The Cancer Genome Atlas database (http://cancergenome.nih.gov/), Molecular Taxonomy of Breast Cancer International Consortium (METABRIC) database (http://www.cbioportal.org/), and GEO database (https://www.ncbi.nlm.nih.gov/geo/).

## Supplementary Materials

Supplementary Materials Figure S1: expression distributions of ZNF genes of patients with breast cancer. Figure S2: the landmark analysis of ZNF341 in radiotherapy patients with TCGA data. Figure S3: GSE31863 data. (a) Hierarchical clustering analysis. The top red and black bands denote the clustered radiosensitive (RS) and radioresistant (RR) patients, respectively. (b–d) The survival curves under radiotherapy and nonradiotherapy for both clustered radiosensitive (RS) and radioresistant (RR) patients. Figure S4: recurrence-free survival stratified by Cox model in GSE31863. (a) RS group. (b) RR group. (c) All radiotherapy patients. RT, radiotherapy; NRT, nonradiotherapy; RS, radiosensitive; RR, radioresistant. Figure S5: coefficient paths for the TCGA data. Figure S6: overall survival stratified by CoxBoost prediction model. The survival curves comparison of radiotherapy and 4-gene-based signature in (a–c) TCGA data and (d–f) METABRIC data. RT, radiotherapy; NRT, nonradiotherapy; RS, radiosensitive; RR, radioresistant. Figure S7: the HR estimation for high expression (High) vs. low expression (Low) of ZNF genes in the CoxBoost prediction model of radiotherapy patients. (a) TCGA data, the adjusted factors are age, pathological stage, histological type, ER, PR, HER, and chemotherapy. (b) METABRIC data, the adjusted factors are age, grade, histological type, ER, PR, HER, and chemotherapy. Figure S8: recurrence-free survival stratified by CoxBoost prediction model in GSE31863. (a) All radiotherapy patients. (b) RS group. (c) RR group. RT, radiotherapy; NRT, nonradiotherapy; RS, radiosensitive; RR, radioresistant. Figure S9: a random forests prediction model. (a) Model prediction error rate for different numbers of survival trees. The prediction results of the random survival forest model on the (b) TCGA dataset, (c) METABRIC dataset, and (d) GSE31863. Figure S10: TCGA data. (a) Hierarchical clustering analysis based on 31 genes. The top red and black bands denote the clustered radiosensitive (RS) and radioresistant (RR) patients, respectively. (b–d) The survival curves under radiotherapy and nonradiotherapy for both clustered radiosensitive (RS) and radioresistant (RR) patients. Figure S11: METABRIC data. (a) Hierarchical clustering analysis based on 31 genes. The top red and black bands denote the clustered radiosensitive (RS) and radioresistant (RR) patients, respectively. (b–d) The survival curves under radiotherapy and nonradiotherapy for both clustered radiosensitive (RS) and radioresistant (RR) patients. Figure S12: overall survival stratified by RSI. The survival curves comparison of radiotherapy and 4-gene-based signature in (a–c) TCGA data and (d–f) METABRIC data. RT, radiotherapy; NRT, nonradiotherapy; RS, radiosensitive; RR, radioresistant. Table S1: univariate and multivariate Cox regression analysis of breast cancer clinical indicators and total survival in the TCGA data. Table S2: univariate and multivariate Cox regression analysis of breast cancer clinical indicators and total survival in the METABRIC data. Table S3: univariate Cox regression analysis of radiotherapy patients and nonradiotherapy patients in ZNF genes high/low expression subgroups in the TCGA data. Table S4: univariate Cox regression analysis of ZNF genes expression and overall survival in the radiotherapy patients in the TCGA data. Table S5: univariate Cox regression analysis of radiotherapy patients and nonradiotherapy patients in ZNF genes high/low expression subgroups in the METABRIC data. Table S6: multivariate Cox regression analysis of ZNF644, ZNF341, ZNF541, and ZNF653 genes in radiotherapy patients with TCGA data.
