# Supplementary material for: Developing ZNF Gene Signatures Predicting Radiosensitivity of Patients with Breast Cancer
Source: J Oncol. 2021 Aug 31;2021:9255494. doi: 10.1155/2021/9255494 (PMC8423582; doi:10.1155/2021/9255494)
Supplement: Supplementary Materials — Figure S1: expression distributions of ZNF genes of patients with breast cancer. Figure S2: the landmark analysis of ZNF341 in radiotherapy patients with TCGA data. Figure S3: GSE31863 data. (a) Hierarchical clustering analysis. The top red and black bands denote the clustered radiosensitive (RS) and radioresistant (RR) patients, respectively. (b–d) The survival curves under radiotherapy and nonradiotherapy for both clustered radiosensitive (RS) and radioresistant (RR) patients. Figure S4: recurrence-free survival stratified by Cox model in GSE31863. (a) RS group. (b) RR group. (c) All radiotherapy patients. RT, radiotherapy; NRT, nonradiotherapy; RS, radiosensitive; RR, radioresistant. Figure S5: coefficient paths for the TCGA data. Figure S6: overall survival stratified by CoxBoost prediction model. The survival curves comparison of radiotherapy and 4-gene-based signature in (a–c) TCGA data and (d–f) METABRIC data. RT, radiotherapy; NRT, nonradiotherapy; RS, radiosensitive; RR, radioresistant. Figure S7: the HR estimation for high expression (High) vs. low expression (Low) of ZNF genes in the CoxBoost prediction model of radiotherapy patients. (a) TCGA data, the adjusted factors are age, pathological stage, histological type, ER, PR, HER, and chemotherapy. (b) METABRIC data, the adjusted factors are age, grade, histological type, ER, PR, HER, and chemotherapy. Figure S8: recurrence-free survival stratified by CoxBoost prediction model in GSE31863. (a) All radiotherapy patients. (b) RS group. (c) RR group. RT, radiotherapy; NRT, nonradiotherapy; RS, radiosensitive; RR, radioresistant. Figure S9: a random forests prediction model. (a) Model prediction error rate for different numbers of survival trees. The prediction results of the random survival forest model on the (b) TCGA dataset, (c) METABRIC dataset, and (d) GSE31863. Figure S10: TCGA data. (a) Hierarchical clustering analysis based on 31 genes. The top red and black bands denote the clustered radiosensitive ( [file 9255494.f1.doc]

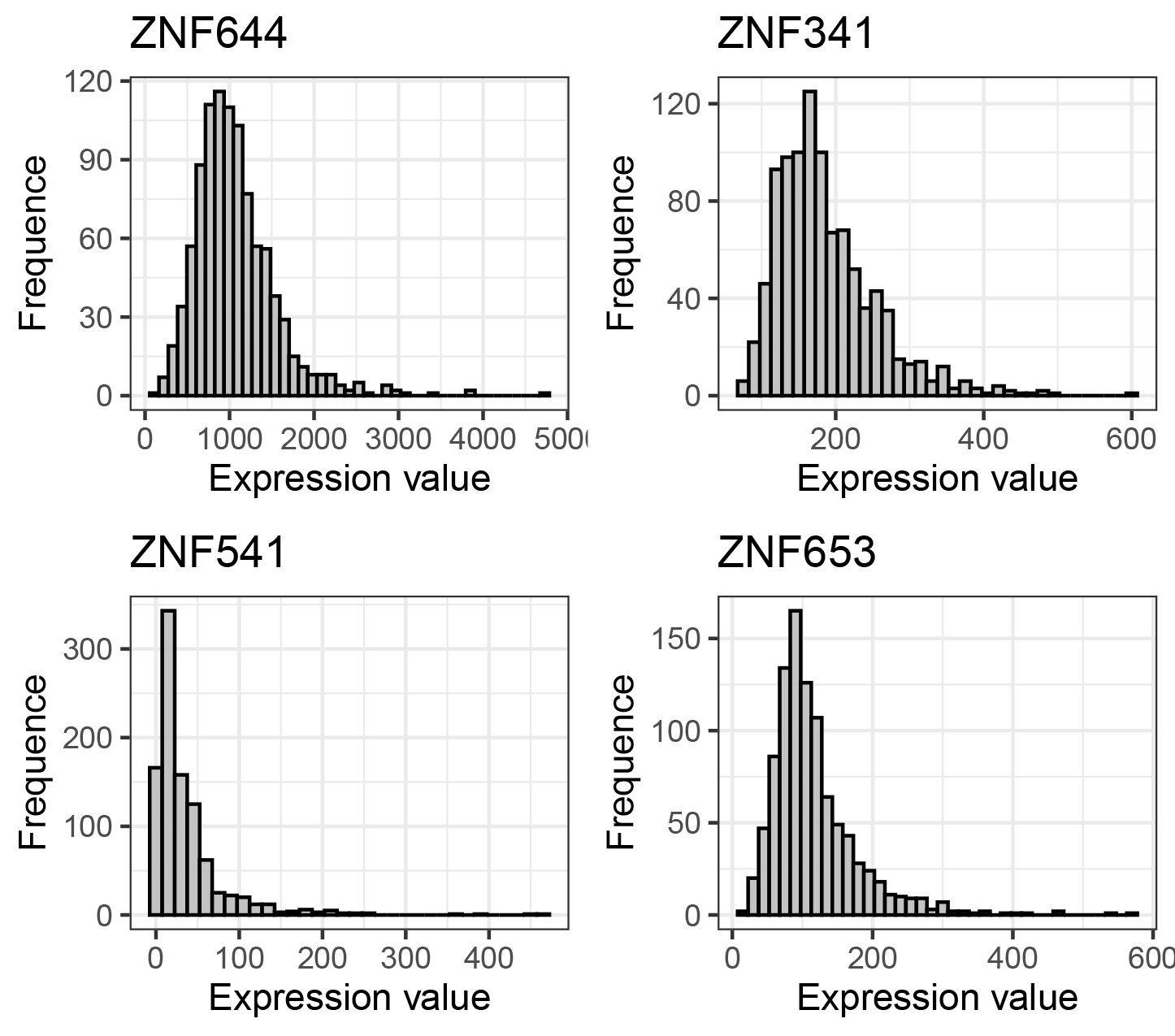


Figure S1**.** Expression distributions of ZNF genes of patients with breast cancer.


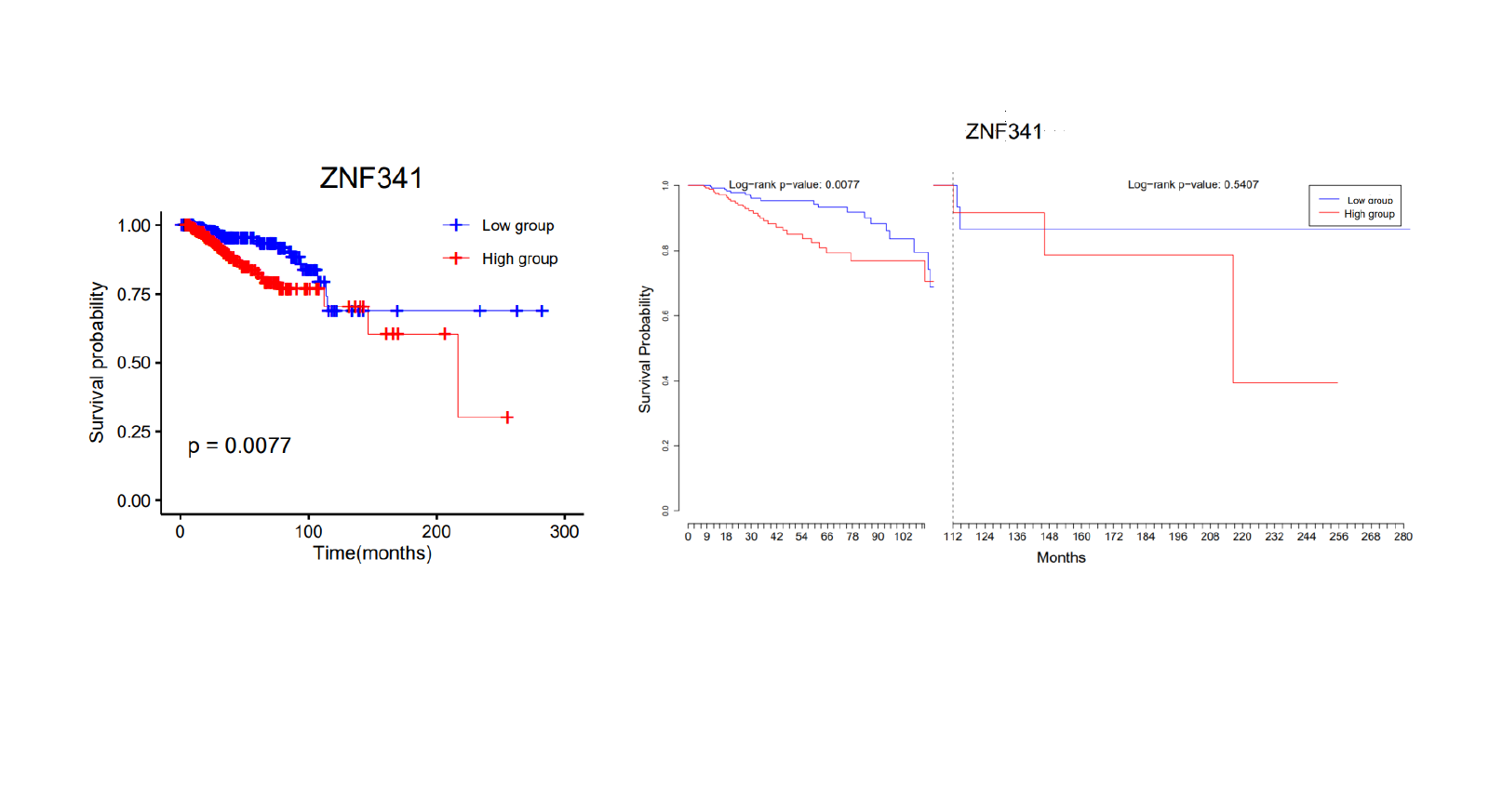


Figure S2. The landmark analysis of ZNF341 in radiotherapy patients with TCGA data.


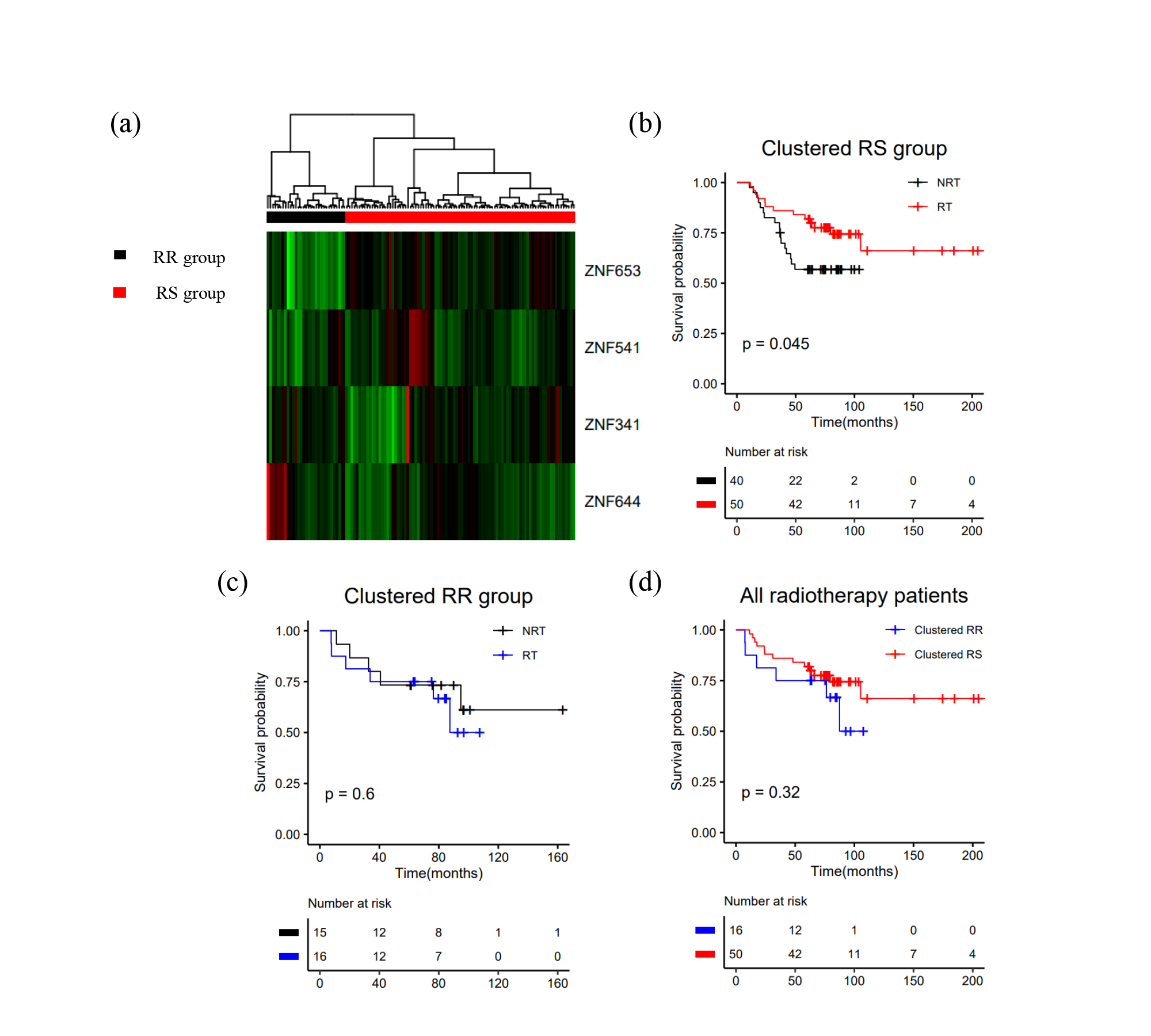


Figure S3. GSE31863 data. (a) Hierarchical clustering analysis. The top red and black bands denote the clustered radiosensitive (RS) and radioresistant (RR) patients, respectively. (b-d) The survival curves under radiotherapy and non-radiotherapy for both clustered radiosensitive (RS) and radioresistant (RR) patients.


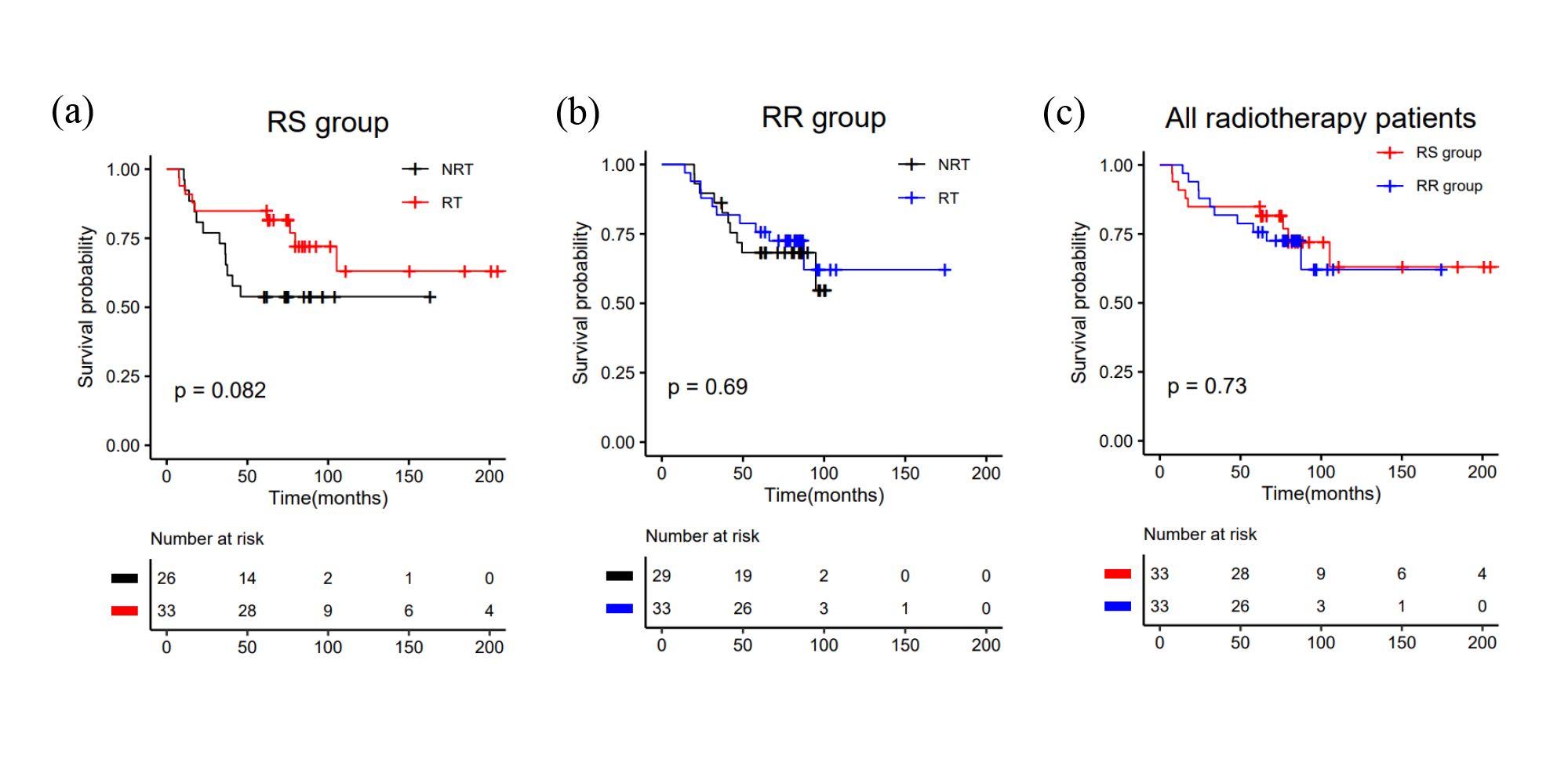
 Figure S4. Recurrence-free survival stratified by Cox model in GSE31863. (a) RS group. (b)RR group. (c) all radiotherapy patients. RT, radiotherapy; NRT, non-radiotherapy; RS, radiosensitive; RR, radioresistant.


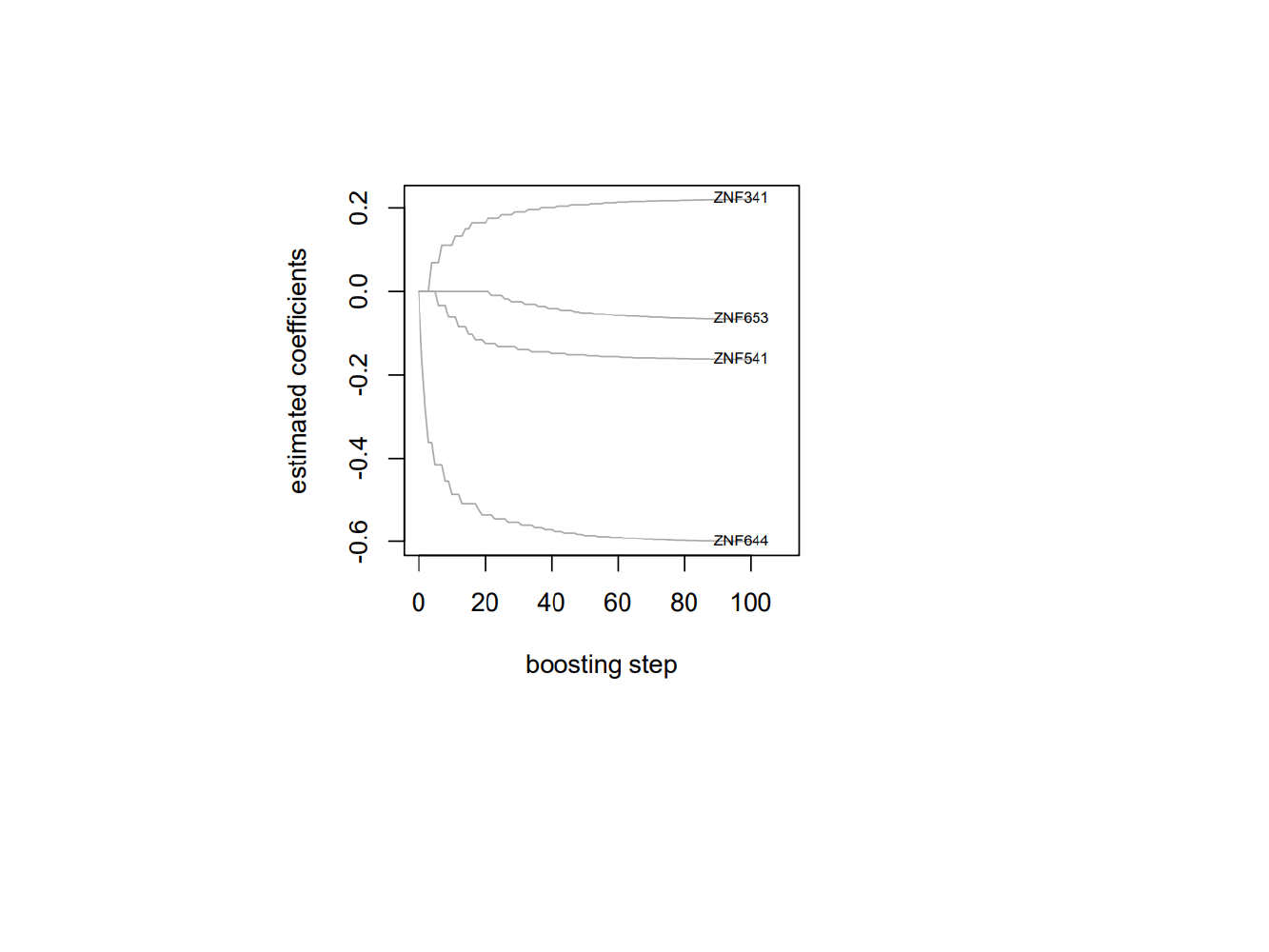


Figure S5. Coefficient paths for the TCGA data.


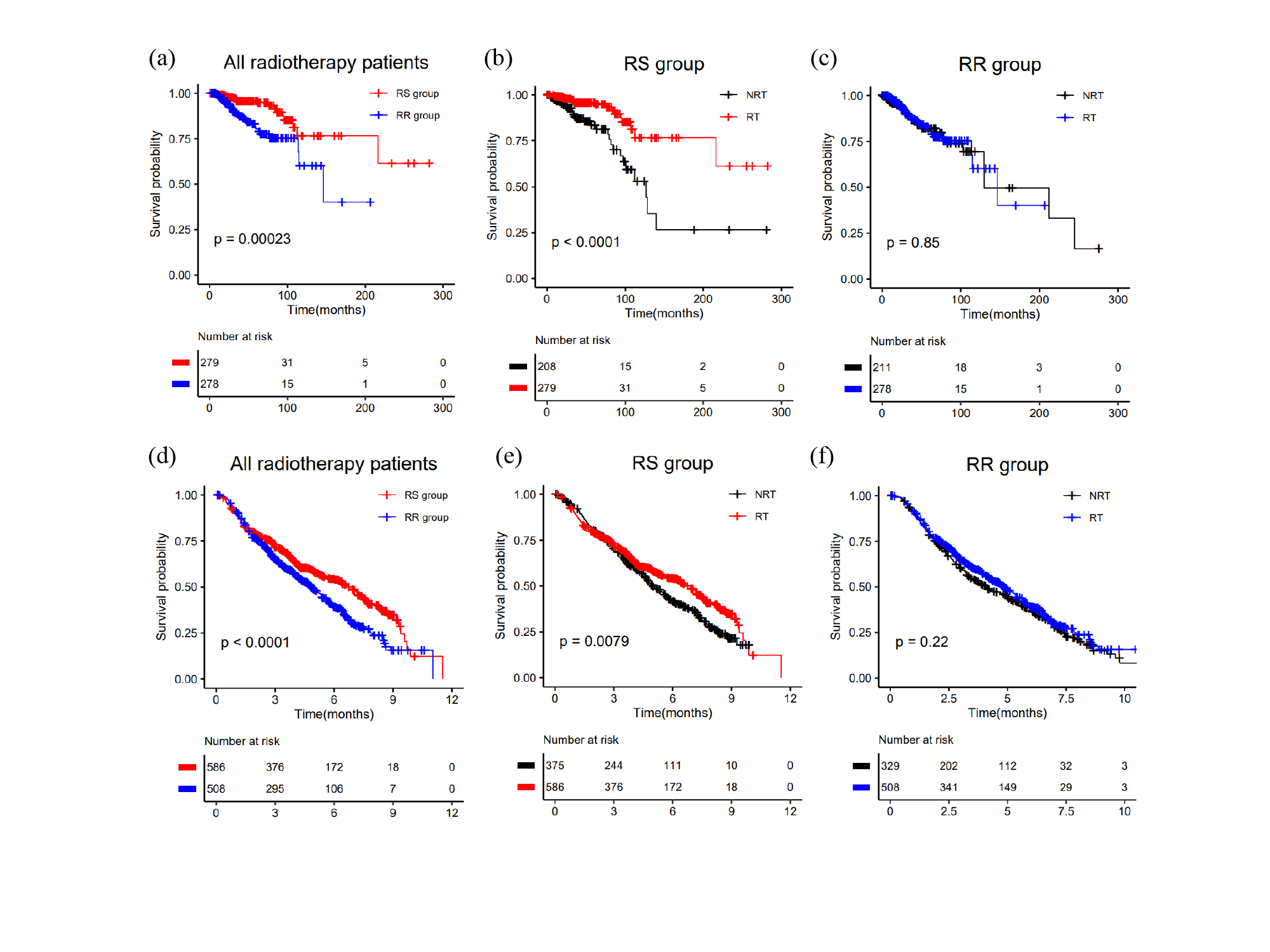


Figure S6. Overall survival stratified by CoxBoost prediction model. The survival curves comparison of radiotherapy and 4-gene based signature in (a-c) TCGA data and (d-f) METABRIC data. RT, radiotherapy; NRT, non-radiotherapy; RS, radiosensitive; RR, radioresistant.


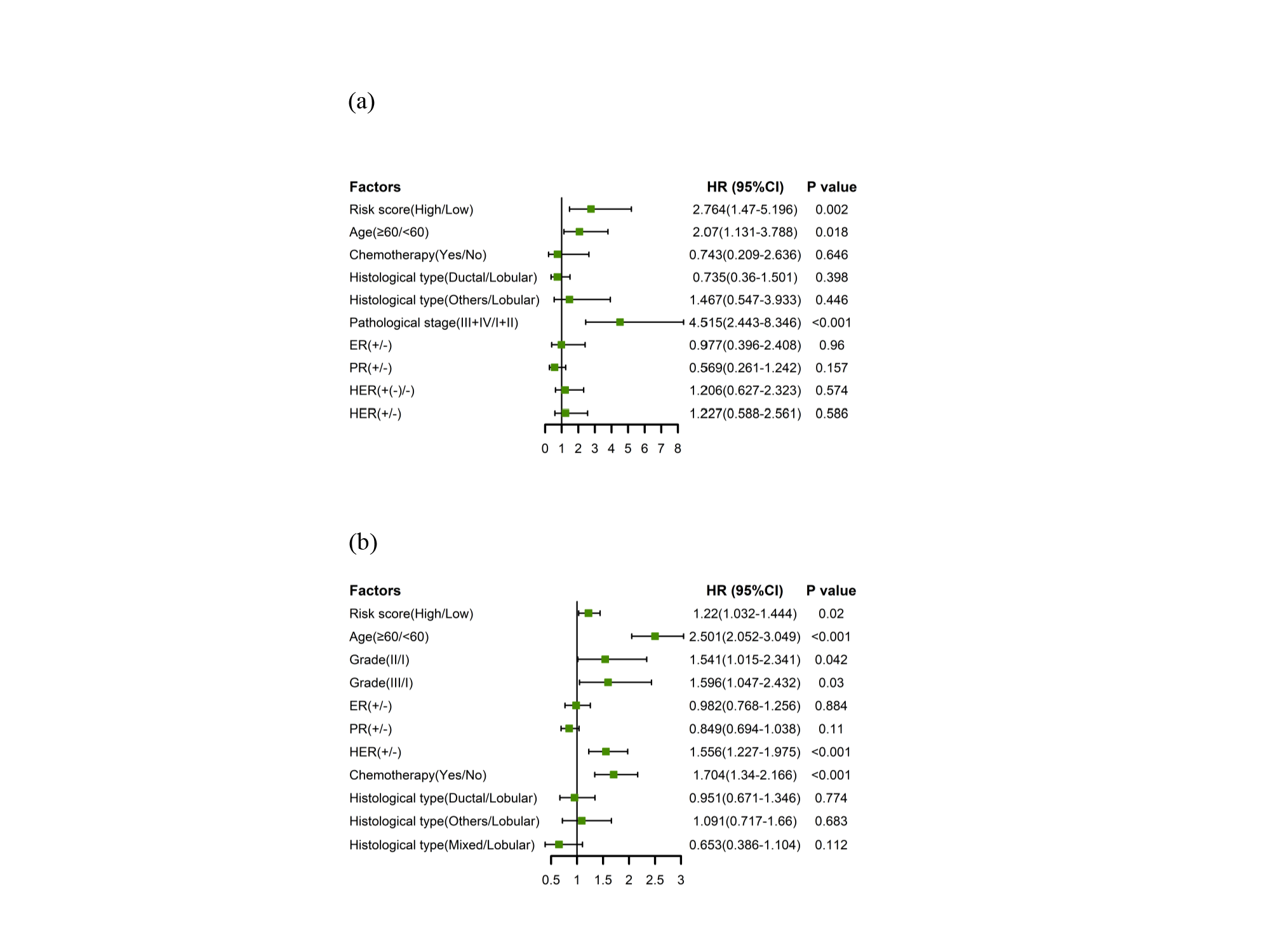


Figure S7. The HR estimation for high expression (High) vs. low expression (Low) of ZNF genes in the CoxBoost prediction model of radiotherapy patients. (a) TCGA data, the adjusted factors are age, pathological stage, histological type, ER, PR, HER and chemotherapy. (b) METABRIC data, the adjusted factors are age, grade, histological type, ER, PR, HER and chemotherapy.


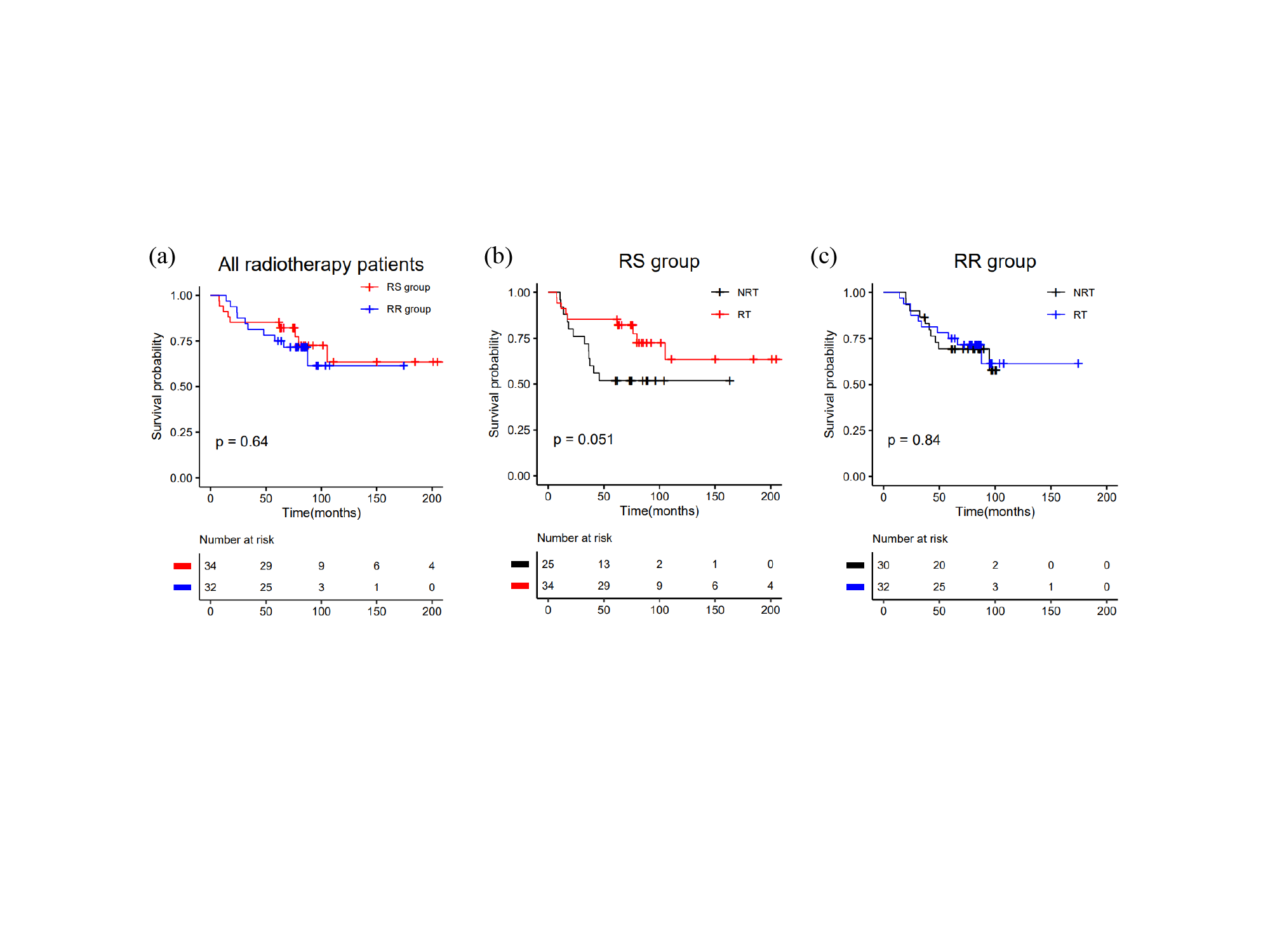
Figure S8. Recurrence-free survival stratified by CoxBoost prediction model in GSE31863. (a) all radiotherapy patients (b) RS group. (c)RR group. RT, radiotherapy; NRT, non-radiotherapy; RS, radiosensitive; RR, radioresistant.


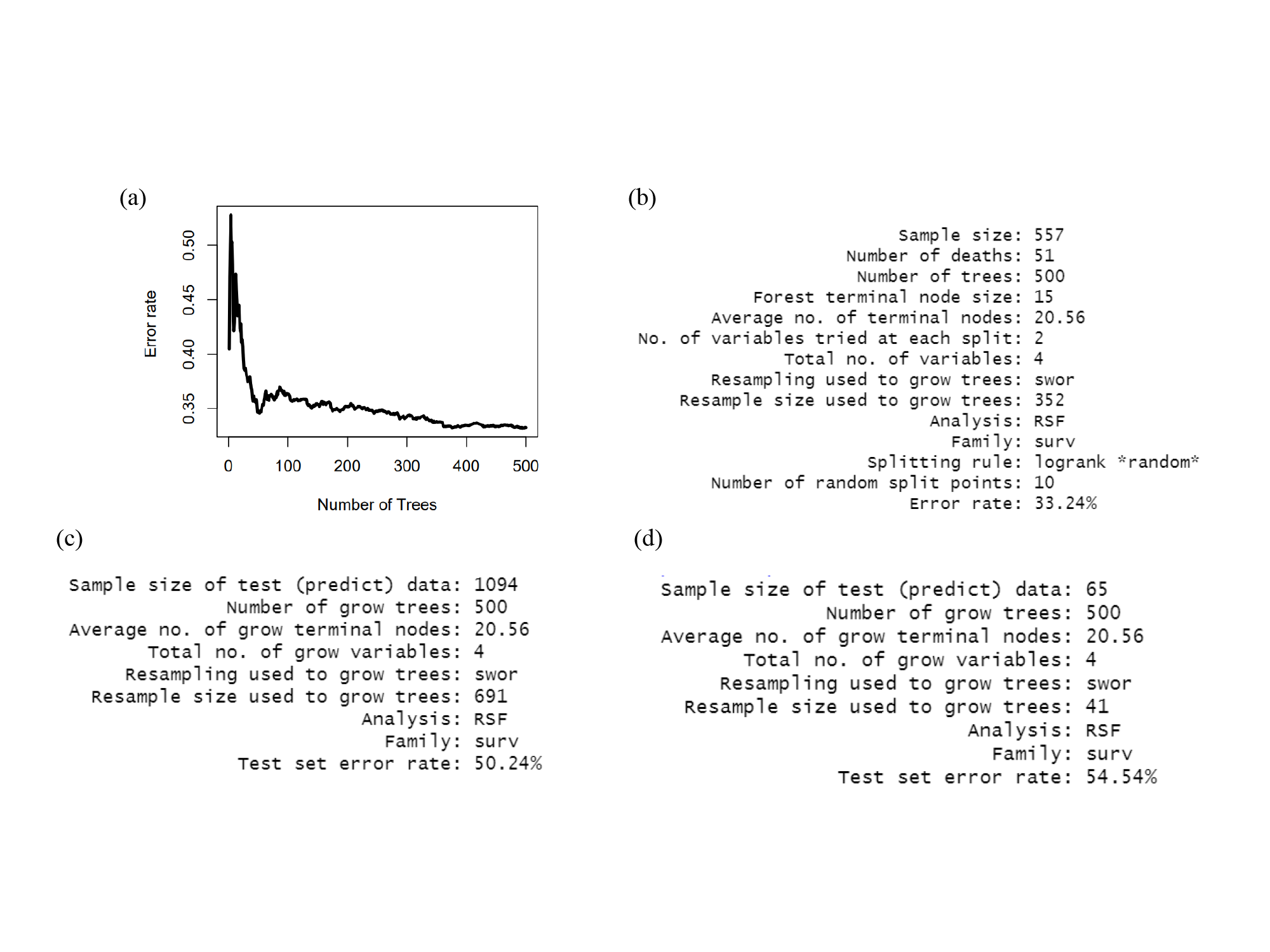


Figure S9. Random forests Prediction Model. (a) Model prediction error rate for different numbers of survival trees. The prediction results of the random survival forest model on the (b)TCGA dataset (c) METABRIC dataset (d) GSE31863.


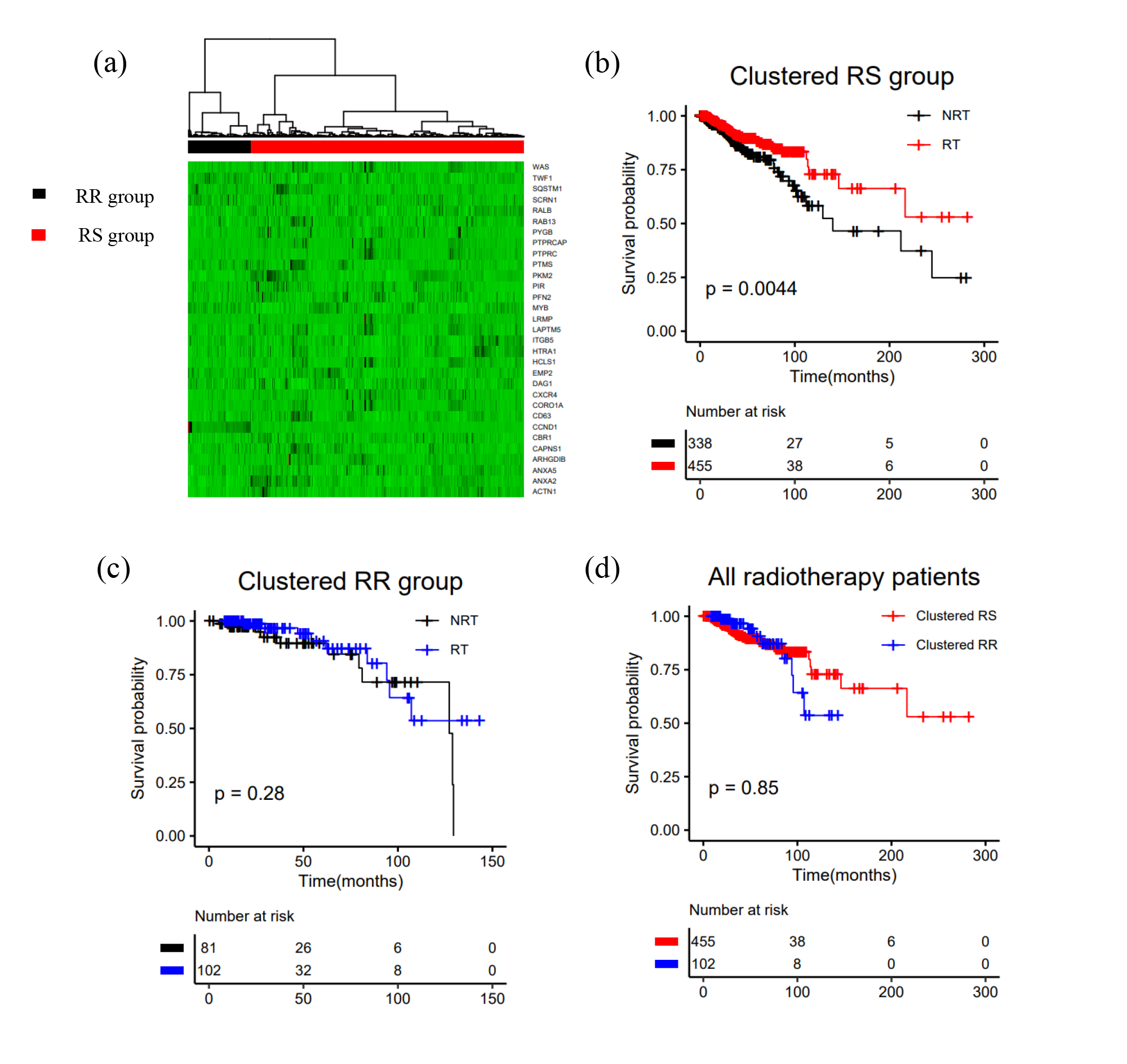


Figure S10. TCGA data. (a) Hierarchical clustering analysis based 31-gene. The top red and black bands denote the clustered radiosensitive (RS) and radioresistant (RR) patients, respectively. (b-d) The survival curves under radiotherapy and non-radiotherapy for both clustered radiosensitive (RS) and radioresistant (RR) patients.


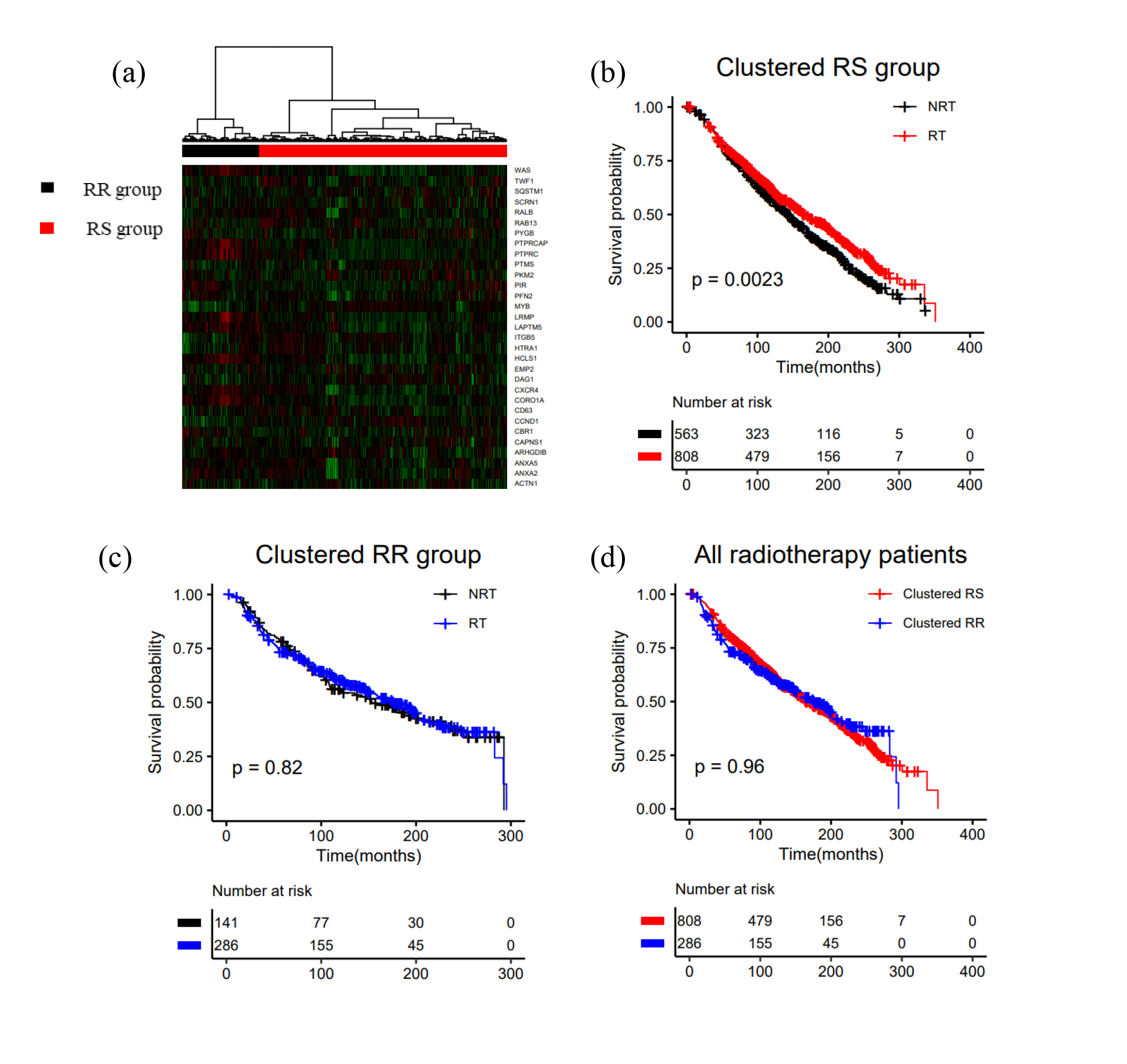


Figure S11. METABRIC data. (a) Hierarchical clustering analysis based 31-gene. The top red and black bands denote the clustered radiosensitive (RS) and radioresistant (RR) patients, respectively. (b-d) The survival curves under radiotherapy and non-radiotherapy for both clustered radiosensitive (RS) and radioresistant (RR) patients.


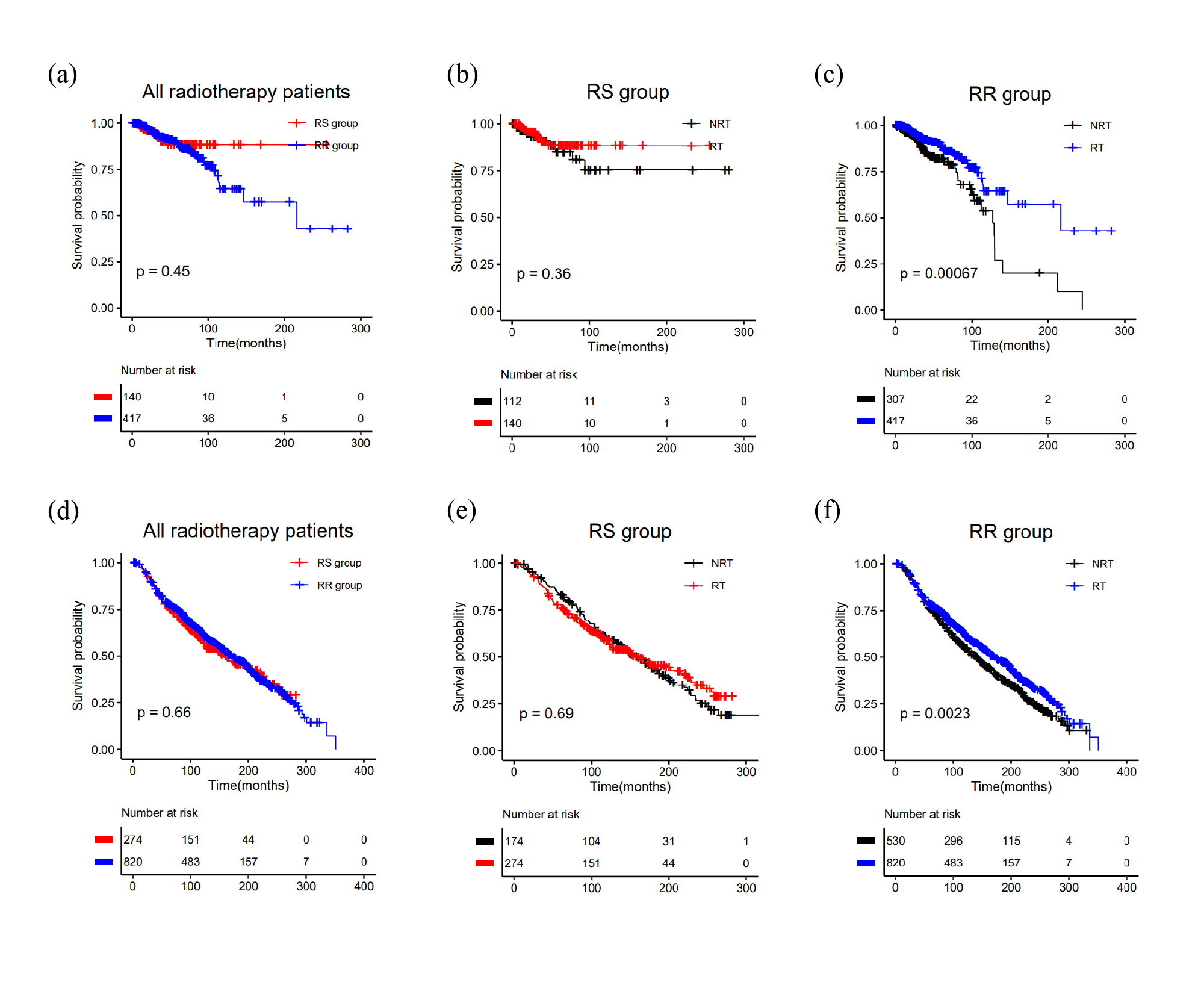


Figure S12. Overall survival stratified by RSI. The survival curves comparison of radiotherapy and 4-gene based signature in (a-c) TCGA data and (d-f) METABRIC data. RT, radiotherapy; NRT, non-radiotherapy; RS, radiosensitive; RR, radioresistant.

Table S1. Univariate and multivariate cox regression analysis of breast cancer clinical indicators and total survival in the TCGA data.

|  | N | Univariate analysis | | Multivariate analysis | |
| --- | --- | --- | --- | --- | --- |
|  | 976 | HR (95%CI) | p | HR (95%CI) | p |
| Age |  |  |  |  |  |
| <60 | 524 | 1.000 |  | 1.000 |  |
| ≥60 | 452 | 2.062(1.405-3.026） | <0.001 | 2.619(1.536-4.464) | <0.001 |
| Histological type |  |  |  |  |  |
| Lobular Carcinoma | 190 | 1.000 |  | 1.000 |  |
| Ductal Carcinoma | 696 | 0.937(0.576-1.527） | 0.795 | 1.243(0.641-2.411) | 0.523 |
| Others | 89 | 1.285(0.659-2.506） | 0.462 | 1.636(0.634-4.224) | 0.314 |
| NA | 1 |  |  |  |  |
| Pathological stage |  |  |  |  |  |
| I/II | 715 | 1.000 |  | 1.000 |  |
| III/IV | 241 | 3.037(2.041-4.520） | <0.001 | 5.345(3.093-9.235) | <0.001 |
| NA | 20 |  |  |  |  |
| ER |  |  |  |  |  |
| ER- | 214 | 1.000 |  | 1.000 |  |
| ER+ | 720 | 0.783(0.504-1.214） | 0.274 | 0.850(0.389-1.858) | 0.685 |
| NA | 42 |  |  |  |  |
| PR |  |  |  |  |  |
| PR- | 307 | 1.000 |  | 1.000 |  |
| PR+ | 624 | 0.721(0.485-1.073） | 0.107 | 0.590(0.290-1.199) | 0.151 |
| NA | 45 |  |  |  |  |
| HER |  |  |  |  |  |
| HER- | 494 | 1.000 |  | 1.000 |  |
| HER+/- | 183 | 0.977(0.535-1.786） | 0.940 | 1.100(0.590-2.050) | 0.766 |
| HER+ | 142 | 1.427(0.754-2.698） | 0.274 | 1.123(0.564-2.234) | 0.743 |
| NA | 157 |  |  |  |  |
| Radiotherapy |  |  |  |  |  |
| No | 419 | 1.000 |  | 1.000 |  |
| Yes | 557 | 0.559(0.383-0.815） | 0.003 | 0.598(0.348-1.028) | 0.068 |
| Chemotherapy |  |  |  |  |  |
| No | 108 | 1.000 |  | 1.000 |  |
| Yes | 778 | 0.469(0.256-0.859） | 0.014 | 0.301(0.145-0.625) | 0.025 |
| NA | 90 |  |  |  |  |

HR: hazard ratio; 95% CI, 95% confidence interval; NA, data not available; ER, estrogen receptor; PR, progesterone receptor; HER, human epidermal growth factor receptor.

Table S2. Univariate and multivariate cox regression analysis of breast cancer clinical indicators and total survival in the METABRIC data.

|  | N | Univariate analysis | | Multivariate analysis | |
| --- | --- | --- | --- | --- | --- |
|  | 1798 | HR (95%CI) | P value | HR (95%CI) | P value |
| Age |  |  |  |  |  |
| <60 | 801 | 1 |  |  |  |
| ≥60 | 997 | 1.961(1.720-2.235) | <0.001 | 2.371(2.043-2.751) | <0.001 |
| Histological type |  |  |  |  |  |
| Lobular Carcinoma | 131 | 1 |  | 1 |  |
| Ductal Carcinoma | 1388 | 0.995(0.790-1.254) | 0.965 | 0.933(0.737-1.179) | 0.559 |
| Mixed | 199 | 0.950(0.716-1.261) | 0.724 | 1.009(0.758-1.342) | 0.953 |
| Others | 80 | 0.557(0.373-0.834) | 0.004 | 0.574(0.382-0.861) | 0.007 |
| Grade |  |  |  |  |  |
| I | 162 | 1 |  |  |  |
| II | 730 | 1.278(0.997-1.637) | 0.052 | 1.146(0.893-1.471) | 0.284 |
| III | 906 | 1.618(1.269-2.063) | <0.001 | 1.332(1.03-1.721) | 0.029 |
| ER |  |  |  |  |  |
| ER- | 425 | 1 |  |  |  |
| ER+ | 1373 | 0.834(0.721-0.963) | 0.014 | 1.015(0.838-1.228) | 0.881 |
| PR |  |  |  |  |  |
| PR- | 856 | 1 |  |  |  |
| PR+ | 942 | 0.764(0.676-0.863) | <0.001 | 0.917(0.791-1.062) | 0.248 |
| HER |  |  |  |  |  |
| HER- | 1574 | 1 |  |  |  |
| HER+ | 224 | 1.530(1.281-1.828) | <0.001 | 1.437(1.192-1.733) | <0.001 |
| Radiotherapy |  |  |  |  |  |
| No | 704 | 1 |  |  |  |
| Yes | 1094 | 0.838(0.741-0.948) | 0.005 | 0.812(0.714-0.923) | 0.002 |
| Chemotherapy |  |  |  |  |  |
| No | 1417 | 1 |  |  |  |
| Yes | 381 | 1.226(1.051-1.430) | 0.009 | 1.806(1.485-2.196) | <0.001 |

HR: hazard ratio; 95% CI, 95% confidence interval; ER, estrogen receptor; PR, progesterone receptor; HER, human epidermal growth factor receptor.

Table S3. Univariate and multivariate cox regression analysis of breast cancer clinical indicators and total survival in the GSE31683 data.

|  | N | Univariate analysis | | Multivariate analysis | |
| --- | --- | --- | --- | --- | --- |
|  | 121 | HR (95%CI) | P value | HR (95%CI) | P value |
| Age |  |  |  |  |  |
| <60 | 69 | 1 |  |  |  |
| ≥60 | 52 | 0.388(0.190-0.793) | 0.009 | 0.594(0.242-1.457) | 0.264 |
| Grade |  |  |  |  |  |
| 1 | 42 | 1 |  |  |  |
| 2 | 40 | 3.201(1.335-7.675) | 0.009 | 2.187(0.860-5.560) | 0.112 |
| 3 | 30 | 3.007(1.181-7.655) | 0.021 | 1.058(0.314-3.568) | 0.928 |
| NA | 9 |  |  |  |  |
| ER |  |  |  |  |  |
| ER- | 29 | 1 |  |  |  |
| ER+ | 92 | 0.292(0.157-0.542) | <0.001 | 0.284(0.094-0.858) | 0.034 |
| PR |  |  |  |  |  |
| PR- | 45 | 1 |  |  |  |
| PR+ | 68 | 0.605(0.325-1.124) | 0.112 | 1.011(0.383-2.669) | 0.983 |
| NA | 8 |  |  |  |  |
| menopause status |  |  |  |  |  |
| pre | 37 | 1 |  |  |  |
| post | 83 | 0.377(0.203-0.699) | 0.002 | 0.484(0.212-1.106) | 0.095 |
| NA | 1 |  |  |  |  |
| Radiotherapy |  |  |  |  |  |
| No | 55 | 1 |  |  |  |
| Yes | 66 | 0.617(0.332-1.148) | 0.127 | 0.456(0.229-0.908) | 0.003 |

HR: hazard ratio; 95% CI, 95% confidence interval; ER, estrogen receptor; PR, progesterone receptor.

Table S4**.** Univariate cox regression analysis of radiotherapy patients and non-radiotherapy patients in ZNF genes high/low expression subgroups in the TCGA data.

| gene names | Low Expression Subgroup  (RT vs Non-RT) | | | High Expression Subgroup  (RT vs Non-RT) | | |
| --- | --- | --- | --- | --- | --- | --- |
|  | HR (95%CI) | P Value | P.adjust | HR (95%CI) | P Value | P.adjust |
| ZNF107 | 0.68(0.419-1.103） | 0.118 | 0.229 | 0.422(0.229-0.78） | 0.006 | 0.025 |
| ZNF138 | 0.679(0.414-1.116） | 0.127 | 0.215 | 0.393(0.215-0.72） | 0.002 | 0.022 |
| ZNF18 | 0.742(0.441-1.251） | 0.263 | 0.227 | 0.407(0.227-0.728） | 0.002 | 0.022 |
| ZNF197 | 0.752(0.449-1.261） | 0.28 | 0.214 | 0.382(0.214-0.681） | 0.001 | 0.017 |
| ZNF221 | 0.808(0.488-1.337） | 0.406 | 0.198 | 0.358(0.198-0.646） | 0.001 | 0.016 |
| ZNF223 | 0.71(0.429-1.175） | 0.183 | 0.22 | 0.403(0.22-0.737） | 0.003 | 0.022 |
| ZNF225 | 0.771(0.475-1.253） | 0.294 | 0.195 | 0.366(0.195-0.686） | 0.002 | 0.019 |
| ZNF227 | 0.687(0.423-1.116） | 0.129 | 0.217 | 0.411(0.217-0.776） | 0.006 | 0.025 |
| ZNF26 | 0.81(0.494-1.326） | 0.402 | 0.179 | 0.336(0.179-0.632） | 0.001 | 0.016 |
| ZNF267 | 0.701(0.434-1.133） | 0.147 | 0.214 | 0.4(0.214-0.745） | 0.004 | 0.022 |
| ZNF268 | 0.704(0.424-1.168） | 0.174 | 0.243 | 0.433(0.243-0.773） | 0.005 | 0.023 |
| ZNF280B | 0.733(0.445-1.209） | 0.224 | 0.23 | 0.412(0.23-0.74） | 0.003 | 0.022 |
| ZNF283 | 0.711(0.427-1.185） | 0.191 | 0.234 | 0.424(0.234-0.768） | 0.005 | 0.023 |
| ZNF284 | 0.711(0.434-1.163） | 0.174 | 0.231 | 0.429(0.231-0.798） | 0.008 | 0.028 |
| ZNF398 | 0.898(0.527-1.529） | 0.691 | 0.198 | 0.346(0.198-0.606） | <0.001 | 0.013 |
| ZNF410 | 0.651(0.399-1.062） | 0.085 | 0.232 | 0.425(0.232-0.779） | 0.006 | 0.024 |
| ZNF420 | 0.852(0.506-1.432） | 0.545 | 0.181 | 0.326(0.181-0.587） | <0.001 | 0.013 |
| ZNF445 | 0.824(0.507-1.339） | 0.434 | 0.174 | 0.328(0.174-0.616） | 0.001 | 0.016 |
| ZNF45 | 0.765(0.466-1.256） | 0.29 | 0.186 | 0.347(0.186-0.648） | 0.001 | 0.016 |
| ZNF460 | 0.652(0.398-1.068） | 0.09 | 0.277 | 0.501(0.277-0.908） | 0.023 | 0.046 |
| ZNF461 | 0.717(0.435-1.179） | 0.19 | 0.204 | 0.374(0.204-0.684） | 0.001 | 0.018 |
| ZNF544 | 0.779(0.468-1.296） | 0.336 | 0.197 | 0.356(0.197-0.642） | 0.001 | 0.016 |
| ZNF561 | 0.725(0.435-1.209） | 0.217 | 0.224 | 0.397(0.224-0.705） | 0.002 | 0.019 |
| ZNF566 | 0.775(0.473-1.268） | 0.31 | 0.184 | 0.339(0.184-0.622） | <0.001 | 0.016 |
| ZNF567 | 0.613(0.374-1.005） | 0.053 | 0.274 | 0.497(0.274-0.899） | 0.021 | 0.044 |
| ZNF569 | 0.667(0.407-1.094） | 0.109 | 0.242 | 0.441(0.242-0.803） | 0.007 | 0.028 |
| ZNF585A | 0.847(0.511-1.404） | 0.519 | 0.168 | 0.309(0.168-0.569） | <0.001 | 0.013 |
| ZNF585B | 0.726(0.447-1.181） | 0.197 | 0.205 | 0.38(0.205-0.704） | 0.002 | 0.021 |
| ZNF597 | 0.773(0.459-1.301） | 0.332 | 0.23 | 0.405(0.23-0.71） | 0.002 | 0.019 |
| ZNF644 | 1.059(0.617-1.817） | 0.835 | 0.164 | 0.29(0.164-0.515） | <0.001 | 0.011 |
| ZNF654 | 0.696(0.418-1.158） | 0.163 | 0.241 | 0.429(0.241-0.761） | 0.004 | 0.022 |
| ZNF655 | 0.649(0.392-1.073） | 0.092 | 0.261 | 0.468(0.261-0.839） | 0.011 | 0.033 |
| ZNF669 | 0.826(0.486-1.405） | 0.482 | 0.204 | 0.362(0.204-0.644） | 0.001 | 0.016 |
| ZNF675 | 0.649(0.399-1.057） | 0.082 | 0.25 | 0.457(0.25-0.835） | 0.011 | 0.033 |
| ZNF717 | 0.774(0.469-1.28） | 0.318 | 0.198 | 0.361(0.198-0.657） | 0.001 | 0.016 |
| ZNF770 | 0.826(0.495-1.378） | 0.463 | 0.186 | 0.337(0.186-0.609） | <0.001 | 0.016 |
| ZNF776 | 0.704(0.426-1.162） | 0.17 | 0.217 | 0.398(0.217-0.727） | 0.003 | 0.022 |
| ZNF341 | 0.439(0.24-0.804） | 0.008 | 0.413 | 0.673(0.413-1.096） | 0.111 | 0.137 |
| ZNF346 | 0.277(0.152-0.506） | <0.001 | 0.592 | 1.024(0.592-1.77） | 0.933 | 0.933 |
| ZNF444 | 0.411(0.226-0.749） | 0.004 | 0.435 | 0.711(0.435-1.163） | 0.174 | 0.199 |
| ZNF503 | 0.362(0.201-0.652） | 0.001 | 0.458 | 0.77(0.458-1.292） | 0.321 | 0.339 |
| ZNF513 | 0.312(0.175-0.555） | <0.001 | 0.551 | 0.933(0.551-1.581） | 0.798 | 0.803 |
| ZNF534 | 0.402(0.217-0.747） | 0.004 | 0.443 | 0.719(0.443-1.167） | 0.182 | 0.207 |
| ZNF541 | 0.313(0.176-0.556） | <0.001 | 0.536 | 0.901(0.536-1.515） | 0.694 | 0.702 |
| ZNF646 | 0.456(0.253-0.821） | 0.009 | 0.404 | 0.667(0.404-1.102） | 0.114 | 0.139 |
| ZNF653 | 0.356(0.194-0.653） | 0.001 | 0.446 | 0.743(0.446-1.237） | 0.253 | 0.277 |
| ZNF668 | 0.298(0.157-0.568） | <0.001 | 0.503 | 0.82(0.503-1.337） | 0.427 | 0.441 |
| ZNF688 | 0.339(0.186-0.62） | <0.001 | 0.513 | 0.839(0.513-1.372） | 0.484 | 0.496 |
| ZNF74 | 0.371(0.201-0.683） | 0.001 | 0.477 | 0.781(0.477-1.28） | 0.327 | 0.344 |
| ZNF75D | 0.451(0.25-0.814） | 0.008 | 0.356 | 0.59(0.356-0.976） | 0.04 | 0.063 |
| ZNF775 | 0.443(0.242-0.812） | 0.008 | 0.417 | 0.685(0.417-1.125） | 0.135 | 0.16 |

RT, radiotherapy; Non-RT, non-radiotherapy. HR: hazard ratio; 95% CI, 95% confidence interval.

Table S5. Univariate cox regression analysis of ZNF genes expression and overall survival in the radiotherapy patients in the TCGA data.

| Gene names | Radiotherapy for all patients （High vs Low） | |
| --- | --- | --- |
|  | HR (95%CI) | P value |
| ZNF107 | 0.489(0.275-0.87) | 0.015 |
| ZNF138 | 0.473(0.264-0.848) | 0.012 |
| ZNF18 | 0.545(0.307-0.969) | 0.039 |
| ZNF197 | 0.493(0.278-0.876) | 0.016 |
| ZNF221 | 0.501(0.283-0.884) | 0.017 |
| ZNF223 | 0.498(0.278-0.894) | 0.02 |
| ZNF225 | 0.443(0.245-0.803) | 0.007 |
| ZNF227 | 0.429(0.237-0.778) | 0.005 |
| ZNF26 | 0.391(0.217-0.705) | 0.002 |
| ZNF267 | 0.405(0.226-0.727) | 0.002 |
| ZNF268 | 0.554(0.315-0.972) | 0.04 |
| ZNF280B | 0.519(0.295-0.914) | 0.023 |
| ZNF283 | 0.525(0.294-0.936) | 0.029 |
| ZNF284 | 0.553(0.312-0.978) | 0.042 |
| ZNF398 | 0.503(0.284-0.889) | 0.018 |
| ZNF410 | 0.539(0.304-0.954) | 0.034 |
| ZNF420 | 0.496(0.277-0.889) | 0.019 |
| ZNF445 | 0.401(0.222-0.727) | 0.003 |
| ZNF45 | 0.441(0.244-0.8) | 0.007 |
| ZNF460 | 0.569(0.325-0.999) | 0.049 |
| ZNF461 | 0.487(0.272-0.873) | 0.016 |
| ZNF544 | 0.511(0.29-0.9) | 0.02 |
| ZNF561 | 0.571(0.327-0.999) | 0.049 |
| ZNF566 | 0.512(0.286-0.917) | 0.024 |
| ZNF567 | 0.567(0.324-0.995) | 0.048 |
| ZNF569 | 0.559(0.313-0.998) | 0.049 |
| ZNF585A | 0.485(0.268-0.877) | 0.017 |
| ZNF585B | 0.475(0.265-0.851) | 0.012 |
| ZNF597 | 0.548(0.313-0.958) | 0.035 |
| ZNF644 | 0.407(0.227-0.728) | 0.002 |
| ZNF654 | 0.547(0.31-0.966) | 0.038 |
| ZNF655 | 0.514(0.292-0.905) | 0.021 |
| ZNF669 | 0.546(0.306-0.973) | 0.04 |
| ZNF675 | 0.528(0.299-0.933) | 0.028 |
| ZNF717 | 0.501(0.28-0.898) | 0.02 |
| ZNF770 | 0.497(0.278-0.891) | 0.019 |
| ZNF776 | 0.528(0.298-0.935) | 0.029 |
| ZNF341 | 2.136(1.206-3.781) | 0.009 |
| ZNF346 | 2.43(1.329-4.444) | 0.004 |
| ZNF444 | 1.929(1.091-3.409) | 0.024 |
| ZNF503 | 1.894(1.057-3.394) | 0.032 |
| ZNF513 | 2.057(1.155-3.662) | 0.014 |
| ZNF534 | 2.274(1.269-4.076) | 0.006 |
| ZNF541 | 1.862(1.054-3.289) | 0.032 |
| ZNF646 | 2.068(1.175-3.638) | 0.012 |
| ZNF653 | 1.984(1.094-3.6) | 0.024 |
| ZNF668 | 2.894(1.561-5.367) | 0.001 |
| ZNF688 | 2.336(1.312-4.158) | 0.004 |
| ZNF74 | 2.184(1.217-3.919) | 0.009 |
| ZNF75D | 1.929(1.088-3.418) | 0.024 |
| ZNF775 | 1.835(1.033-3.259) | 0.038 |

HR: hazard ratio; 95% CI, 95% confidence interval.

Table S6. Univariate cox regression analysis of radiotherapy patients and non-radiotherapy patients in ZNF genes high/low expression subgroups in the METABRIC data.

| Gene names | Low Expression Subgroup  (RT vs Non-RT) | | High Expression Subgroup  (RT vs Non-RT) | | Radiotherapy for all patients  (High vs Low) | |
| --- | --- | --- | --- | --- | --- | --- |
|  | HR (95%CI) | P Value | HR (95%CI) | P Value | HR (95%CI) | P value |
| ZNF18 | 0.906(0.758-1.082） | 0.275 | 0.755(0.635-0.897） | 0.001 | 0.745(0.631-0.878） | <0.001 |
| ZNF644 | 0.848(0.716-1.005） | 0.057 | 0.830(0.694-0.993） | 0.042 | 0.795(0.675-0.938） | 0.006 |
| ZNF717 | 0.939(0.792-1.114） | 0.473 | 0.733(0.614-0.876） | 0.001 | 0.835(0.709-0.984） | 0.032 |
| ZNF341 | 0.763(0.633-0.918） | 0.004 | 0.906(0.768-1.068） | 0.238 | 1.538(1.303-1.816） | <0.001 |
| ZNF503 | 0.788(0.659-0.943） | 0.009 | 0.902(0.762-1.069） | 0.235 | 1.259(1.069-1.483） | 0.006 |
| ZNF541 | 0.786(0.655-0.944） | 0.01 | 0.902(0.764-1.065） | 0.222 | 1.274(1.081-1.501） | 0.004 |
| ZNF653 | 0.806(0.670-0.970） | 0.023 | 0.865(0.734-1.02） | 0.085 | 1.251(1.061-1.475） | 0.008 |

RT, radiotherapy; Non-RT, non-radiotherapy; HR: hazard ratio; 95% CI, 95% confidence interval

Table S7. Multivariate cox regression analysis of ZNF644, ZNF341, ZNF541, ZNF653gene in radiotherapy patients with TCGA data.

| Gene names | Coefficients | HR (95%CI) | P value |
| --- | --- | --- | --- |
| ZNF644 | -0.61414 | 0.541(0.354-0.827) | 0.005 |
| ZNF341 | 0.21667 | 1.242(0.931-1.657) | 0.141 |
| ZNF541 | -0.14907 | 0.862(0.676-1.097) | 0.227 |
| ZNF653 | -0.06819 | 0.934(0.673-1.297) | 0.684 |

HR, hazard ratio; 95% CI, 95% confidence interval.
